# Zinc–Bromine Rechargeable Batteries: From Device Configuration, Electrochemistry, Material to Performance Evaluation

**DOI:** 10.1007/s40820-023-01174-7

**Published:** 2023-08-31

**Authors:** Norah S. Alghamdi, Masud Rana, Xiyue Peng, Yongxin Huang, Jaeho Lee, Jingwei Hou, Ian R. Gentle, Lianzhou Wang, Bin Luo

**Affiliations:** 1 Australian Institute for Bioengineering and Nanotechnology (AIBN), The University of Queensland, Brisbane, QLD 4072 Australia; 2 School of Chemistry and Molecular Biosciences, Faculty of Science, The University of Queensland, Brisbane, QLD 4072 Australia; 3 School of Chemical Engineering, The University of Queensland, Brisbane, QLD 4072 Australia; 4 Department of Chemistry, Faculty of Science, Imam Mohammad Ibn Saud Islamic University (IMSIU), 11564 Riyadh, Saudi Arabia

**Keywords:** Zinc–bromine rechargeable batteries, Cell configurations, Electrochemical property, Performance metrics, Assessment methods

## Abstract

Zinc–bromine rechargeable batteries (ZBRBs) are one of the most powerful candidates for next-generation energy storage due to their potentially lower material cost, deep discharge capability, non-flammable electrolytes, relatively long lifetime and good reversibility. However, many opportunities remain to improve the efficiency and stability of these batteries for long-life operation. Here, we discuss the device configurations, working mechanisms and performance evaluation of ZBRBs. Both non-flow (static) and flow-type cells are highlighted in detail in this review. The fundamental electrochemical aspects, including the key challenges and promising solutions, are discussed, with particular attention paid to zinc and bromine half-cells, as their performance plays a critical role in determining the electrochemical performance of the battery system. The following sections examine the key performance metrics of ZBRBs and assessment methods using various ex situ and in situ/operando techniques. The review concludes with insights into future developments and prospects for high-performance ZBRBs.

## Highlights

- A comprehensive discussion of the recent advances in zinc–bromine rechargeable batteries with flow or non-flow electrolytes is presented.
- The fundamental electrochemical aspects including the key challenges and promising solutions in both zinc and bromine half-cells are reviewed.
- The key performance metrics of ZBRBs and assessment methods using various ex situ and in situ/operando techniques are also discussed.

## Graphical abstract

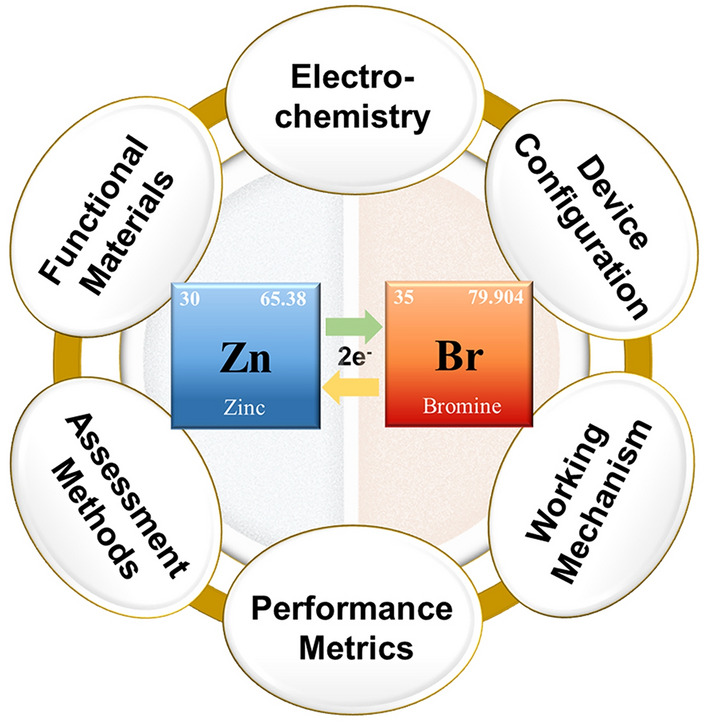

## Introduction

Increasing environmental and energy crises around the globe have prompted the growing demand for clean and renewable energy [1–6]. Close to 40% of the world’s electricity will be produced from renewable sources by 2030 [7]. Solar photovoltaics and wind power generators have become more popular over the past decade, but their inherently intermittent, fluctuating nature makes it challenging to integrate them into existing electrical grid systems [1, 8]. Energy storage systems (ESSs) that are safe, cost-efficient and reliable have been developed to satisfy the surge in demand for green electricity. Several characteristics make electrochemical energy storage devices excellent candidates, including their ability to combine power and energy, and their geographic flexibility, compact design and scalable construction and installation [9]. These systems also provide input stabilisation, high-power charging, load shifting and an uninterruptible power supply and are found to be useful in many other applications [1, 10]. While lithium-ion rechargeable batteries dominate the current market for grid-scale electrochemical energy storage devices, they have different limitations, including relatively low power density, high cost for replacement and maintenance and flammable organic electrolytes. Therefore, a reliable, energy-efficient, eco-friendly and affordable ESS should be developed to accelerate the transition from fossil fuels to renewable energy with clean technologies on a global scale.

Zinc–bromine rechargeable batteries (ZBRBs) are regarded as one of the most promising devices for use in emergency uninterruptible power supplies and load levelling for grid-scale stationary power applications due to their long lifecycle, high safety, sustainability, high theoretical energy density, low cost and the wide availability of active materials [11]. In brief, ZBRBs are rechargeable batteries in which the electroactive species, composed of zinc–bromide, are dissolved in an aqueous electrolyte solution known as redox (for reduction and oxidation), which can potentially convert chemical energy into electricity when needed under controlled conditions. The water-based electrolytes in ZBRB systems make them less prone to overheating and causing fires than batteries with highly flammable electrolytes (e.g. lithium-ion batteries). In the early stage of zinc–bromine batteries, electrodes were immersed in a non-flowing solution of zinc–bromide that was developed as a flowing electrolyte over time. Both the zinc–bromine static (non-flow) system and the flow system share the same electrochemistry, albeit with different features and limitations. All details provided herein will pertain to both static and flow ZBRBs unless otherwise specified.

The balance between the cost of active materials and the cost of balance-of-plant components is a significant challenge with all ESSs. While the cost of the active materials can be reduced through using inexpensive materials, the cost of other components in the system (e.g. tanks, pumps, control system) can offset these savings and lead to a higher cost for the system as a whole. Therefore, it is important to consider not only the cost of the active materials but also the system’s total cost to ensure the successful implementation of ESSs. In addition to the cost, the system’s lifecycle and performance are critical factors that must be considered [11]. Zinc–bromine flow batteries have shown promise in their long cycle life with minimal capacity fade, but no single battery type has met all the requirements for successful ESS implementation. Achieving a balance between the cost, lifetime and performance of ESSs can make them economically viable for different applications.

ZBRBs are categorised as hybrid batteries which means that some of the energy is stored at the negative electrode (anode) via metallic zinc plated during the charging phase, while the remaining energy is stored in a liquid phase at the catholyte. It is important to note that the size and density of the plated Zn and the catholyte storage tank determine the storage capacity of ZBRB systems, resulting in power rating and capacity that correspond to each other. Typically, the practical implementations of ZnBr_2_ systems are challenged by: (1) zinc dendrite growth resulting from repeated electroplating and stripping of zinc that can pierce the membrane and eventually forms a conductive bridge between the electrodes (shorting), (2) hydrogen gas generation as the electrochemical potential of charge/discharge process of the system which is higher than that required for water hydrolysis which competes with the reduction reaction of Zn^2+^ ions and decreases the overall efficiency of the ZBRBs, (3) corrosive elemental bromine liquid, Br_2_(l), production at the positive electrode during charge, which can be diffused through the membrane to the zinc half-cell reacting with the Zn plated at the negative electrode (crossover), causing self-discharge and/or degradation, and (4) the low miscibility (~ 2.8 vol%) and stratification behaviour of Br_2(l)_ in aqueous solutions that can lead to non-uniform concentration distributions [12]. Overcoming these challenges can be achieved by improving the suitable configurations of ZBRBs, understanding the cell chemistry and attributes and selecting appropriate assessment methods. All these aspects will be highlighted in more detail in the following sections.

## Cell Configurations of Zinc–Bromine Rechargeable Batteries

### Static (Non-flow) Configurations

**Fig. 1:**
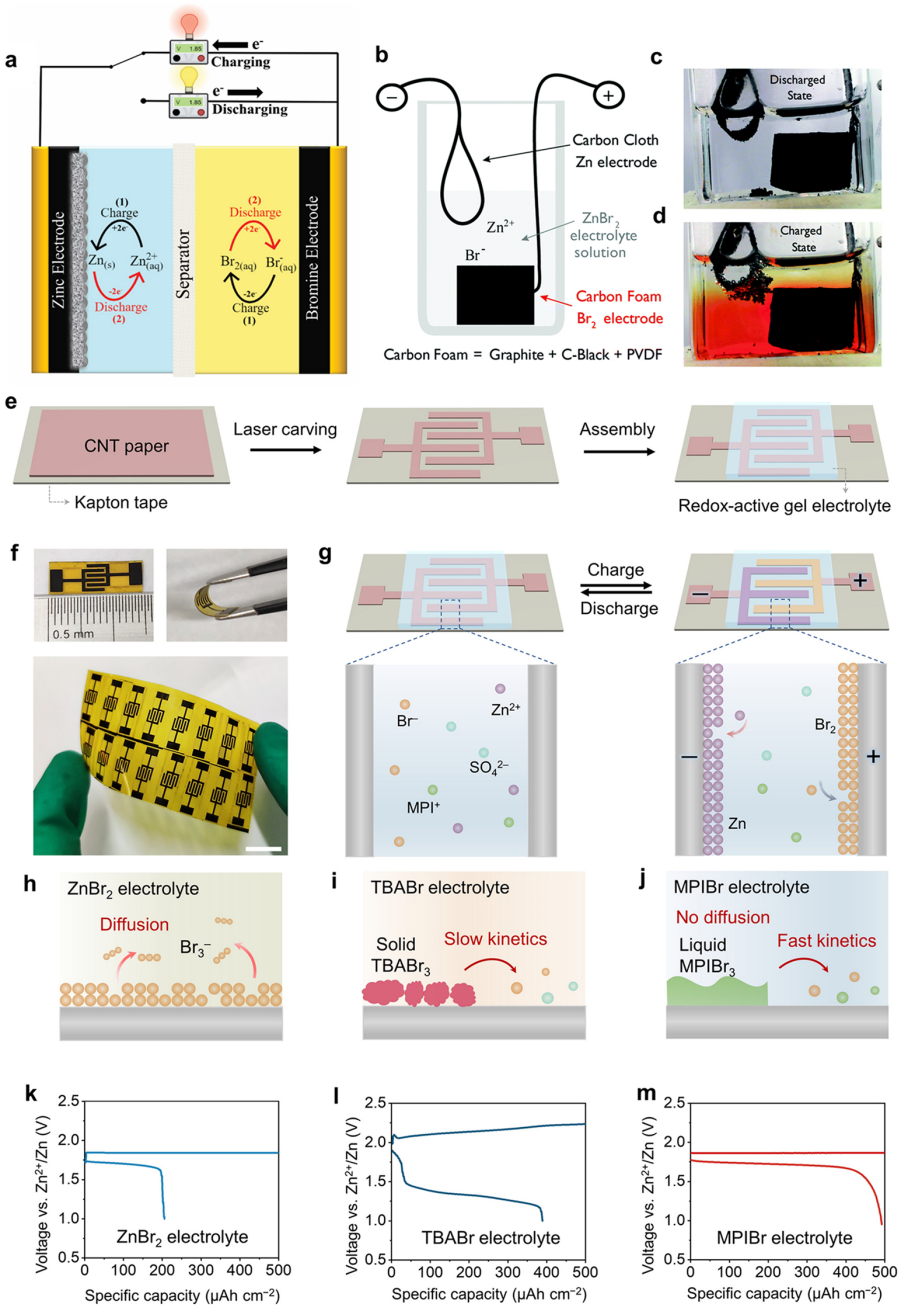
Schematic representation of different static cells. **a** ZBRB with static non-flow configuration. **b** MA-ZBB cell design schematic. The photographs of the realised 5 mL cell in the **c** discharged and **d** charged states show the distinct colours of Br_2(l)_ (red), dissolved Br_2_(aq) (yellow) and ZnBr_2_(aq) electrolyte (transparent). Panels **b**–**d** reproduced with permission from Ref. [12]. Copyright 2017, The Royal Society of Chemistry. **e** Fabrication process of the ZnBr_2_ MBs. **f** Digital photographs of flexible Zn–Br_2_ MBs at flat and bending states. **g** In situ construction of Br_2_ cathode and Zn anode during the charging process. **h** Schematic of the fast diffusion of Br_3_^−^ from current collector when ZnBr_2_ solution is used as the electrolyte. **i** When TBABr is used as the electrolyte, solid-state TBABr_3_ complex is produced, which shows slow reaction kinetics. **j** When MPIBr is used as the electrolyte, the oily phase MPIBr_3_ complex is generated, which not only prevents the Br_3_^−^ from dissolving into the electrolyte but also shows fast reaction kinetics. Charge–discharge curves of ZBBs with **k** ZnBr_2_, **l** TBABr and **m** MPIBr electrolytes. Panels **e**–**m** reproduced with permission from Ref. [15] Copyright 2022, SCIENCE ADVANCES

Static non-flow zinc–bromine batteries are rechargeable batteries that do not require flowing electrolytes and therefore do not need a complex flow system as shown in Fig. 1a. Compared to current alternatives, this makes them more straightforward and more cost-effective, with lower maintenance requirements. The initial configuration type of zinc–bromine static batteries, which was proposed by Barnartt and Forejt [13], consisted of two carbon electrodes immersed in a static ZnBr_2_ electrolyte and separated by a porous diaphragm. In this design, an activated charcoal layer was pasted on the positive electrode that was vertically oriented in the cells to control the bromine diffusion rate, thus improving charge retention. The static ZBRB is characterised by low weight compared to the flow-type ZBRBs, as it eliminates the need for auxiliary parts (e.g. pumps, tubes, tanks), resulting in higher cost and complicated manufacturing processes. However, its high self-discharge rate and low energy density have hindered development and commercialisation in the past decades [14]. Gao et al. [11] recently demonstrated that the low energy efficiency and high self-discharge rate of zinc–bromine static batteries can be overcome while retaining the electrochemical advantages of zinc–bromine redox couples by using a glass fibre separator. The authors also claimed that using the complexing agent, tetrapropylammonium bromide, could reversibly convert Br_2_/Br_3_^−^ species to solid phases, thus suppressing self-discharge. In addition to providing good interfacial contact, porous carbons also provide physical confinement to prevent cross-diffusion and self-discharge.

Zinc–bromine batteries with gel electrolytes are another type of rechargeable static battery that uses a gel electrolyte to transport ions between the electrodes, eliminating the need for a pump system. The gel electrolyte contains the active materials (zinc and bromine) in a semi-solid texture that provides easy handling and reduces leakage risks. The gel electrolyte provides a three-dimensional aspect to the electrolyte matrix, maintains homogenous conditions and uniform distribution of the active species over the electrode surfaces, resulting in better battery performance [16]. They are also comparatively cheap and are greener with a long cycle life, meaning they can be charged and discharged many times without significant degradation in performance. This indicates that zinc–bromine batteries can gain several advantages with gel electrolytes compared to other types of batteries [16]. The Gelion Endure™ company has developed a zinc–bromine gel electrolyte system that is viable commercially. However, much like all battery types, zinc–bromine batteries with gel electrolytes also have disadvantages. They might be sensitive to temperatures over 50 °C, which requires careful monitoring and management to ensure they operate within their optimal temperature range, while they also require regular maintenance, including monitoring the state of charge and replacing the gel electrolyte periodically.

Researchers have recently proposed innovative strategies to make ESSs more cost-effective and viable by avoiding membranes and flow pumping systems [17–19]. A membraneless, flowless zinc–bromine battery exhibits an extremely low levelised cost of energy stored (LCOES) of $0.29 per kWh per cycle for 1000 cycles in comparison with lithium-ion batteries of about $0.5 per kWh per cycle with a life of ∼ 1500 cycles and an average LCOES of $0.75 per kWh per cycle for advanced lead-acid batteries with a limited cycle life [17]. Interestingly, membraneless, flowless ZBBs could achieve an average LCOES of less than $0.01 per KWh per cycle if the cell lasted for 10,000+, competing with ZBFBs with an average LCOES of less than $0.1 per KWh per cycle with more than 10,000 cycles. However, these outcomes are still proposed and under investigation. Based on this, no traditional battery can meet the technical cost requirements of ESSs apart from membraneless and flowless zinc–bromine batteries [12]. This design offers several advantages over conventional flow batteries, including reduced weight, lower cost, and simplified maintenance requirements. Biswas et al. [12] described a low-cost, membraneless, single-chamber, minimal architecture zinc–bromine secondary battery (MA-ZBB) (Fig. 1b–d), which did not require forced convection and utilised liquid bromine and porous carbon foam electrodes as well as allowing zinc dendrites to form freely with enhanced efficiencies. Using a carbon foam electrode, the authors demonstrated the local containment of Br_2_ and explored how colour tracking and feedback monitoring could be used to actively control the transport of reactive species. The cost of each cell was reported to be roughly $94 per kWh including passives, with coulombic and energy efficiencies of 95% and 75%, respectively, over 1000 cycles. The authors stated this non-forced-flowing battery could be attractive due to its cost, lifetime and performance characteristics for grid-scale energy storage applications. While this configuration eliminates the need for a membrane, which can reduce cost and simplify the construction of the battery, it requires more research for careful control of the electrolyte flow and the reaction kinetics necessary for future applications.

Microsized zinc–bromine batteries are another configuration of ZBRBs that operate using the same basic electrochemistry as larger zinc–bromine batteries, but the electrodes and electrolyte are designed to be smaller and more compact. This can be achieved using microfabrication techniques, such as photolithography, which allows for precise control over the size and shape of the battery components. The active zinc and bromine are typically stored in small microscale structures, such as microchannels or microfluidic devices, to minimise the volume and weight of the battery. One potential application for microsized zinc–bromine batteries is in portable electronic devices, such as smartphones and laptops [15]. However, these candidates typically suffer from complicated fabrication procedure and low energy density. Besides, all cathodes of current microbatteries (MBs) are solid state, and the trade-off between areal capacity and reaction kinetics restricts their wide applications [3]. Recent study [15] proposed a dual-plating strategy to facilely prepare zinc–bromine MBs with a liquid cathode for high areal energy density and fast manufacturing procedure. Through using laser carving method, carbon nanotube (CNT) sheet (~ 10 µm thick) was initially adhered to insulating tape and then patterned into interdigitated electrodes (~ 0.18 cm^2^). This microelectrode, that was flexible and easily integrated (Fig. 1e–g), was covered by a redox-active gel electrolyte containing Zn^2+^ and Br^−^ ions over the interdigitated electrodes, where the prepared Zn–Br_2_ MBs were at the discharging state. The authors claimed that using 1-methyl-3-propylimidazolium bromide (MPIBr) complexing agent in these batteries achieved fast redox kinetics of the complex and high Columbic efficiency compared to that with ZnBr_2_ and tetrabutylammonium bromide (TBABr) electrolytes (Fig. 1h–m). Although this research presents new insights for Zn–Br_2_ MBs, further research is needed to promote the reliability and applicability of these configurations for viable commercial applications.

### Flow Configurations

Currently, no technology is available that can satisfy all the exemplary characteristics of an optimal energy storage system for large-scale applications [7]. The redox flow battery (RFB) is among the emerging storage techniques that can hopefully meet these requirements. One of the key advantages of RFBs is their independence from power generation and energy capacity [20–22]. In addition to the amount of electrolyte stored in the battery system, the concentration of active species, the voltage of each cell and the number of stacks in the battery determine the battery's capacity [23, 24]. The power generated, however, depends on the behaviour of the active materials and the electrode size, meaning highly scalable and flexible [23]. Once set up and in a running mode, these systems need low maintenance and little attention because they have few moving parts and require minimal operations [25]. Modular construction is also possible due to its simple design [9]. In theory, RFBs have no limited life cycle because of their simplicity and reversible redox reactions (eliminating solid-state reactions) [26]. It is possible for these batteries to be fully charged and discharged without significant damage to their components [27]. Because of their rapid response times, measured in milliseconds [28], these systems are well suited for levelling intermittent renewable power output. For all these reasons, the flow battery is a promising future energy storage source. Combined with its relatively low operating and capital costs, it becomes a viable alternative to other emerging energy storage systems [27, 29].

RFBs are secondary batteries that perform redox reactions in an electrolyte solution using electrochemically active species [30]. They can be charged by various power sources and then discharged to power external loads. Based on the redox reactions, most RFBs can be classified as all-liquid RFBs and/or solid-hybrid RFBs. RFBs with all-liquid components (e.g. all-vanadium RFBs) are soluble in their electrolytes, whereas solid-hybrid RFBs (e.g. zinc–bromine RFBs) involve a solid plating/stripping process in at least one electrode reaction [31]. All-liquid RFBs allow full decoupling of energy and power but suffer from low energy density [20, 32]. The solid-hybrid RFBs, on the other hand, can achieve high energy density at the cost of design scalability and flexibility [31]. Several RFB systems have been proposed and reviewed [33–40], including the all-vanadium redox flow battery (VRFB) [24, 41, 42], organic RFBs [43–48], iron-chromium flow battery (ICFB) [49–51] and a wide range of zinc-based hybrid RFBS, such as zinc–iron, zinc–cerium, zinc–polymer, zinc–iodine and zinc–bromine [52–58]. Although some of these technologies have been well-developed and commercialised (e.g. VRFB), they suffer from various drawbacks, such as less abundance and high costs [59, 60]. The overall system cost has hampered the market’s adoption of these technologies and increased the need for low-cost redox-active materials, which are naturally abundant to meet the requirements of RFB chemistries. For stationary storage, flow batteries should be compared to more mature technologies as standards before implementation. For instance, typical lead-acid batteries operate within the KW to MW range with intermediate cycle life, cost, and discharge duration [61]. Li-ion batteries are another mature technology that has similar power range, higher cost, and longer cycle life [62]. On the other hand, flow batteries have power ranges from KW to hundreds of MW with high discharge durations, a very long cycle life and competitive costs when it comes to row materials.

The ZBFB has substantial advantages over other flow batteries, such as high energy density, high cell voltage and the low cost of the materials used [63–66]. In general, the positive species in ZBFBs cost far less than those in the zinc–cerium system, and their energy density is three times higher [67]. The carbon electrodes and active electrolytes in a ZBFB cost around $8/kWh [26]. When integrated into a complete system, the cost is approximately $200/kWh, which is still competitive in comparison to the cost of an all-vanadium flow battery ($200–750/kWh) [67]. Another important advantage of the ZBFB as a low-cost EES candidate is its potential cost-effective electrolyte. ZBFBs are expected to incur lower overall production costs due to lower raw material costs than others (e.g. all-vanadium flow batteries). The industrial scale production of bromine and zinc is already well established, where Br_2_ has a spot price of about US$1620 per tonne (from 2006 prices) and zinc is about US$2050 per tonne after inflation adjustment [68]. Thus, ZBFBs are appealing and suitable for storing renewable energy (e.g. solar and wind) driven by their attractive features. In addition, the modular design of ZBFBs makes them one of the most practical types of RFBs for multi-kW and MW scales [63]. Meidensha Electric Co., for example, developed and operated ZBFBs at the MW scale in Japan as one of the advanced battery systems for its Moonlight Project between the 1980s and early 1990s [2], with more than 1300 cycles completed with a 66% energy efficiency in this system [2]. In addition, several companies have operated or developed various field installations of ZBFBs, including Redflow Ltd. [69] and Primus Power [70].

**Fig. 2:**
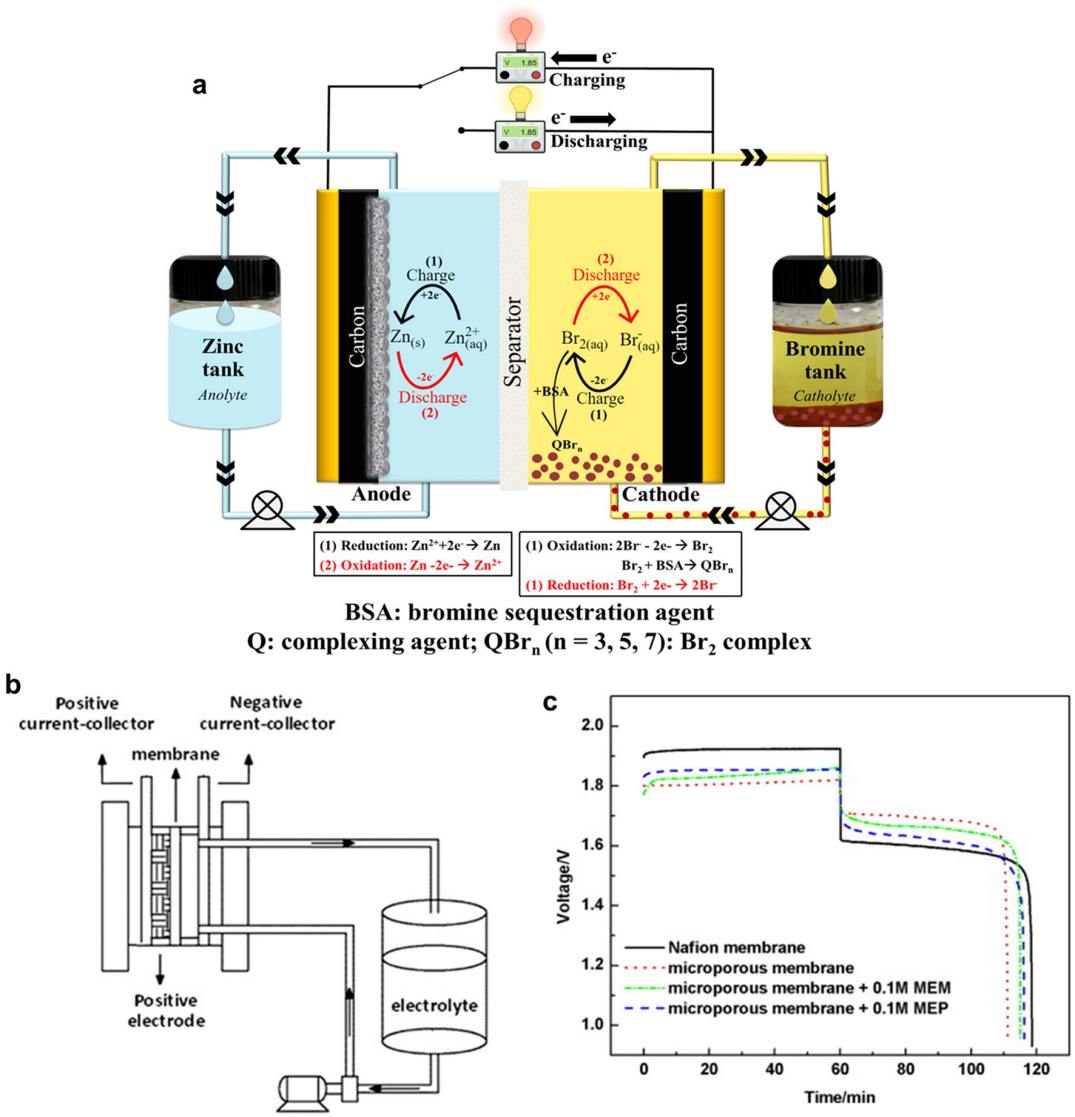
**a** Typical ZBFB with the redox reaction mechanism and different components. **b** Schematic diagram of a single-flow zinc–bromine battery. **c** Charge–discharge curves of single-flow ZBB at room temperature under a constant current density of 20 mA cm^−2^. Panels **b** and **c** reproduced with permission from Ref. [72]. Copyright 2013, Elsevier

The typical ZBFB (Fig. 2a) has two electrodes, a negative electrode (zinc) and a positive electrode (bromine), which are all separated by a membrane to prevent cross-contamination. Two tanks of aqueous electrolyte solutions (anolyte and catholyte) contain electrochemically active species, including zinc (Zn^2+^) and bromide (Br^−^) ions, respectively, where elemental bromine exists in equilibrium with bromide ions forming polybromide ions, Br_*n*_^−^, where *n* = 3, 5 and 7. These tanks are connected to two pumps to circulate the electrolyte solutions over both electrode surfaces, and the electrochemical energy is stored or released during the charge and discharge process of the battery. Despite the fact that pumps increase the complexity of the system, they are necessary for controlling generated heat, feeding and homogenising reactants, removing bromine complexes from the stack and ensuring uniform zinc deposits [71]. However, the flow system (e.g. pump) can affect the battery’s efficiency and parasitic reactions, consuming some of the energy produced by the battery. The flowing electrolytes can also cause corrosion and degradation of the battery components, reducing the battery’s performance and lifetime.

In contrast to the traditional zinc–bromine redox flow batteries, constructed with two heavy electrolyte tanks and pumps that sacrifices some of the energy density, a new system has been proposed with only one tank and pump installed in half of the battery system (Fig. 2b). This configuration can achieve lower weight and cost and thus improve the energy density of the system. The use of a catholyte tank and its pump was avoided by modifying the structure of the bromine electrode. This simple design, with a lower weight and cost, appears more suitable for single-flow batteries, since one part of the electrochemical reactions in hybrid-flow batteries is based on plating and stripping. Lai et al. [72] designed a novel single-flow ZBRB without catholyte tank to simplify the system design, prevent bromine diffusion and improve the energy density of the battery system. In this study, different membranes (Nafion 115, Daramic) and complexation agents [*N*-methyl-ethyl-pyrrolidinium (MEP) and *N*-methyl-ethyl-morpholinium (MEM)] were used to achieve the optimal assembly materials for this configuration. Although the charge/discharge behaviour (Fig. 2c) of the batteries assembled with the microporous membranes presented better performance and lower ohmic resistance, Coulombic efficiency (CE) reduced from 98 to 83% with microporous membrane cells, compared to that with Nafion. This was attributed to the high bromine diffusion through the membrane micropores which could be improved by adding suitable complex agents to the bromine-side slurry made over a piece of carbon felt as a positive electrode of the system. Considering the membrane cost and adding appropriate complexing agents, Daramic microporous membrane was selected as a separator and MEM as a complexation reagent. Consequently, the performance of the fabricated battery could reach 92% CE and 82% energy efficiency (EE) at a constant current density of 20 mA cm^−2^, comparable to the traditional zinc–bromine flow battery. While the new battery design exhibited a lower weight, improved energy density and inhibited bromine diffusion compared to conventional ZBFBs, investigating different properties of various membranes, complexing agents and other modification approaches should be considered to make such configuration reliable for future commercialisation.

Overall, the choice of ESS configuration will depend on the application’s specific requirements, including reliability, performance and scalability. When selecting an ESS, it is important to consider all of these factors and evaluate the options to determine the best fit for the intended application.

## Electrochemistry and Materials for Zinc–Bromine Rechargeable Batteries

### Zinc Half-Cell

In ZBRBs, zinc electrodes undergo reversible plating (deposition) and stripping electrochemical reactions. During charge, ionic Zn^2+^ accepts electrons from the external circuit, and metallic zinc is plated (reduced) as a thick layer on the electrode surface [14, 68, 73]. When the battery is discharged, the plated zinc oxidises (strips) and dissolves in the electrolytes. These reactions are described by the following equations:

1 $${\text{Zn}}^{2 + } + 2e^{ - } \to {\text{Zn}}^{0} \;({\text{Charging}})$$

2 $${\text{Zn}}^{0} \to {\text{Zn}}^{2 + } + 2e^{ - } \;({\text{Discharging}}) \;(E^{^\circ } = - 0.763\;{\text{vs SHE at}}\;25\,{^\circ } {\text{C}})$$

The ZBRB efficiencies can be influenced by the number of plating and stripping processes. Lex and Matthews [71] emphasised the necessity to strip the zinc in ZBRBs for extended periods to ensure a smooth electrode surface for next zinc deposition. The authors stated that the residual zinc left on the anode after discharge results in the loss of 3–5% of the amp-hour capacity. However, the remaining zinc could potentially be used as a useful energy source if additional zinc is plated over it in the subsequent cycles.

The electrochemical performance of the zinc half-cell can be affected by the morphology of zinc electrodeposits produced during the charging phase. The zinc plating structure in the subsequent discharge phase also determines efficiencies, charge densities and peak current values [68]. The quality of zinc plating morphology is influenced by several factors, including electrolyte composition, direct current (DC) flow, exchange current density and operating temperature [74]. A previous study demonstrated that the behaviour and kinetics of zinc cations are strongly affected by other supporting electrolytes in aqueous solutions containing bromide [75]. Significantly, the hydration structure of zinc cations in aqueous solutions plays a critical role when using electrode materials with different properties (e.g. high-porosity materials) on the zinc side. Furthermore, the effect of operating temperature on zinc deposits was investigated by scientists, who stated that the zinc deposits, which were grey in appearance, turned black at temperatures higher than 40 °C, while the authors also reported that smooth and bright zinc deposits were obtained when increasing the electrolyte’s zinc concentration [74].

**Fig. 3:**
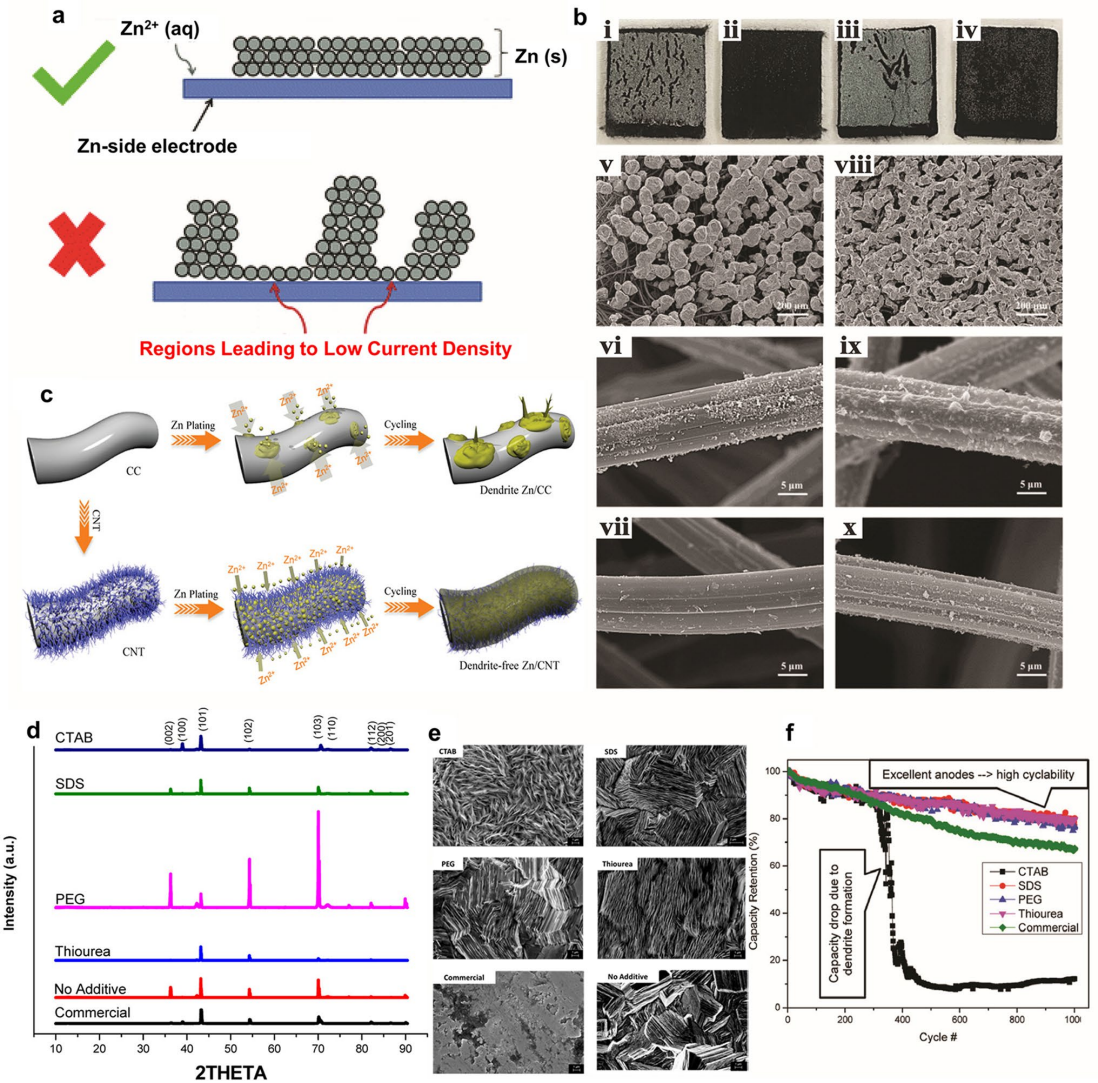
**a** Graphical illustration of how a lower degree of zinc plating uniformity potentially results in lower zinc-side electrode current densities. Reproduced with permission from Ref. [68]. Copyright 2016, Springer. **b** Digital photographs of (i, ii) GF and (iii, iv) thermally treated GF negative electrodes after the charging process; (i) and (iii) are taken from the membrane side, while (ii) and (iv) are taken from the current collector side. SEM images of (v–vii) GF and (viii–x) thermally treated GF negative electrodes following the charge process. The top, medium and bottom parts are taken from the parts on the membrane side, underneath the zinc layer, and on the current collector side, respectively. Reproduced with permission from Ref. [78]. Copyright 2018, Elsevier. **c** Illustration of zinc deposition on carbon cloth (CC) and carbon nanotube (CNT). Reproduced with permission from Ref. [79]. Copyright 2020, Wiley–VCH Verlag GmbH & Co. KGaA, Weinheim. **d** XRD results of the zinc anode electroplated with and without organic additives and commercialised zinc. **e** SEM images of synthesised anode with and without organic additives and commercialised zinc (magnification 5 k). CTAB, SDS, PEG, thiourea: Electroplated Zn using CTAB, SDS, PEG or thiourea containing electrolyte, respectively. Commercial: Commercial zinc foil material. No additive: Electroplated zinc without using any additive in the electroplating bath. **f** Cyclability of the batteries with zinc anode with organic additives and commercialised zinc foil. Estimated errors: ± 2.5%. Panels **d**–**f** reproduced with permission from Ref. [80]. Copyright 2017, American Chemical Society

The activity of zinc electrode reactions primarily entails the formation of dendrites and the shape change of the electrode surface. In the initial stage, zinc deposition begins with nucleation and continues with growth, meaning the formation of dendrites is a cumulative result of battery cycling, not a single cycle. It is more likely that zinc ions will deposit on a zinc nucleus rather than nucleating at a new site since zinc nucleation has a higher overpotential than zinc growth [76]. This can lead to a non-uniform distribution of the active species forming dendrites [77]. When the deposits are uneven, certain regions on the surface are not fully utilised, resulting in low current density, as shown in Fig. 3a. Furthermore, the formation of zinc dendrites over repeated charging process can damage the membrane, causing short circuits and battery self-discharge [67]. Consequently, obtaining suitable zinc plating morphology and inhibiting dendrite growth are important for long cycle life and a high-performance zinc–bromine battery.

A three-category approach to zinc dendrites can be drawn from a variety of proposed zinc dendrite issues, including (1) strategies to prevent the formation and further growth of zinc dendrites as far as possible, (2) strategies to mitigate the adverse effects generated by zinc dendrites and (3) strategies to suppress zinc dendrites and ultimately produce processes without dendrites [81]. Jiang et al. [78] employed a bottom-up approach to investigate specific methods for uniformly distributing zinc in negative electrodes and to promote commercial applications of ZBFBs. In this work, thermal treatment was used to increase the defects of the original graphite felt (GF) electrodes with a few defects. The authors observed that zinc tended to plate on the membrane sides (Fig. 3bi and iii) than on the current collectors (Fig. 3bii and iv), for both original and thermally treated GF, due to lower ionic conductivity. However, a thin layer of zinc plating was detected on the thermally treated electrode than on the original one. At the microscale observation, zinc distribution was highly uniform with a thicker and smoother zinc layer on the thermally treated electrode (Fig. 3bviii–x) indicating that ion transport was highly enhanced by more carbon defects within the porous electrode. This approach was recommended to improve properties of the anode materials and thus the ZBRB performance. However, investigation of the electrochemical performance (e.g. charge–discharge profile) was neglected in this study suggesting more research and electrochemical examinations of such method.

Based on the above-mentioned categories, Table 1 presents various solutions that have been proposed in literature to inhibit zinc dendrite and mitigate its adverse effects in different zinc-based rechargeable batteries which can be considered when developing zinc electrode in ZBRBs [82–92].

**Table 1:** Proposed solutions to addressing zinc dendrite issues in Zn-based rechargeable batteries

Strategies	Materials	Principle	Function	Limitation
Additives into electrolytes	Polymer (PEI, PEG, PVA) Organic molecules (Alcohols, DMSO)	Adsorption on the zinc-based electrode surface	Block the formation sites of zinc dendrites Slow down the depositing rate of zinc	Increase the polarisation of zinc-based electrodes
	Metal ions (Bi^3+^, Pb^2+^)	Substrate effect	Optimise the current distribution of electrodes	Influence the electrochemistry of flow batteries
Additives into electrodes	Polymer (PTFE, PE)	Binder	Reduce the generation sites of dendrites Decrease the solubility of zinc-based compounds	NA
	Metal oxides or metal hydroxides (PbO, In(OH)_3_)	Substrate effect Alloying of Zn-based electrodes	Optimise the current distribution of electrodes Regulate the deposition of Zn^2+^	Influence the FB electrochemistry HER issue of Zn alloy
Modification of the electrode structure	(3D) structure design	Improvement of zinc activity, stability and reversibility	Increasing the surface area of the electrodes to make a uniform deposition of zinc	NA

The interfacial mechanism of zinc deposition plays a key role in designing dendrite-free zinc electrodes. Dendrite growth is accelerated by electrons and ions accumulating at the tips of zinc seeds as the intensity of the electric field and ion concentration increases. A flat electrode surface with uniform nucleation was proposed to minimise the inhomogeneity of zinc growth [93]. In general, there are two interfaces within zinc deposition stages (i.e. nucleation and growth) proposed in zinc-based batteries, the zinc–electrolyte interface and a host–zinc interface, which involve the plating/stripping of zinc on the bare surface during operation and zinc plated on host materials that is used to modify the bare electrode surface, respectively [93]. Understanding the impact of interfacial zinc deposition behaviour is critical to achieving a better performance of zinc electrodes in aqueous systems. Based on the solid electrolyte interphase (SEI) theory, electrons and ions are two key factors affecting zinc deposition and reaction interphases. Due to water decomposition, high zinc deposition reactivity and instability are critical issues in aqueous electrolytes. Therefore, different design strategies should be considered to optimise the SEI and regulate the interfacial electric field for stable zinc deposition and suppressed dendrites. Optimising the aqueous electrolyte formulation is one strategy for reducing surface reactions. Another proposed strategy involves creating an artificial interfacial layer between the zinc and the electrolyte that performs the same function as an SEI [93, 94]. The construction of interfacial layers is primarily accomplished through in situ chemical pre-treatment or ex situ coating. A previous study demonstrated a protection layer made by the atomic layer deposition of TiO_2_ and coating on the zinc electrodes [95]. In this research, zinc corrosion was significantly suppressed, resulting in less gas evolution and Zn(OH)_2_ by-product formation. Zinc plated with reduced gas generation could maintain the effective contact area between electrolyte and anode, improving the CE. A further approach to inhibiting zinc dendrite growth is polymer addition, which can poison active interface sites at the zinc half-cell. Ganne et al. [96] studied an electrolyte containing a long-chain polymer commercial additive and a concentrated chloride electrolyte used to investigate the kinetics of zinc deposition. In this study, the simulations of electrode kinetics revealed that polymer additives alter deposit morphology by changing a number of specific rate steps: slowing down charge transfer reactions, poisoning active interface sites and strengthening hydrogen adsorption. This led to more active sites and intermediate ions on a surface covered with zinc deposition, which could be adapted for ZBRB studies. It is important to note that the most challenging part of SEI formation is the chemical dynamics at the interface during the initial charging and discharging process. Specifically, during the initial charging, the negatively charged anode in the liquid electrolyte could be approached by solvated cations, while the applied negative potential repels anions [97]. The rearrangement of the solvated anions and cations and their enrichment or depletion would form an electric double layer at the SEI, which would occur before the reductive degradation of electrolyte components. Based on its structure and composition, this double layer is expected to significantly influence the subsequent interphase, showing how the electrolyte contributes to the SEI and its durability [97]. However, the experimental proof of such a double layer is still absent in terms of ZBRBs, and its link to interphase chemistry remains merely a theory.

Carbon-based electrodes are commonly used as alternative materials since they have a large specific surface area, good chemical stability and good electrochemical stability [78, 98–100]. Modifying the carbon electrode materials is one of the most common strategies used to achieve uniform zinc morphology and activate the zinc electrode electrochemical reactions, thus improving cell operation efficiency. This can be achieved by increasing the reaction rate via catalysis, increasing the surface area for the reaction to occur and/or altering the nature of the active material in contact with the electrolyte solution. Another method involves improving electrode surface selectivity, which controls species that reach and react with the electrode. Whatever approach is chosen, the goal is to improve zinc deposition and stripping during the charging and discharging processes by significantly increasing the rate of the Zn^2+^*/*Zn charge transfer reaction and reversibility. Zeng et al. [79] achieved dendrite-free plating behaviour using a 3D carbon nanotube framework on a flexible carbon cloth. Scale model simulations following nucleation indicate that these excellent properties can be attributed to small zinc nuclei and homogeneous electric fields, as shown in Fig. 3c. While the zinc dendrite issue on the negative side leads to low power density and poor cycle life in ZBRBs, thus restricting further commercialisation of this system, there have been very few reports discussing how this problem can be solved based on the modifications of carbon-based materials (e.g. carbon felt or graphite felt), and most studies have focused on such a strategy to accelerate the reaction kinetics of the Br_2_/Br^−^ couple.

Zinc anode has demonstrated highly promising characteristics, such as abundance of natural resources, nontoxicity, a high theoretical capacity (820 mAh g^−1^, 5855 mAh cm^3^), a high overpotential for hydrogen (H_2_) evolution, low redox potential (− 0.76 V vs. the standard hydrogen electrode/SHE) and long cycle life [101, 102]. While metallic electrodes also offer low charge transfer resistance, they are costly and subject to severe degradation in aqueous electrolytes from processes such as uncontrolled dendrite formation, corrosion and dissolution, which negatively affects the long-term performance and operation of ZBRBs [103–106]. Sun et al. [80] examined the suppression of dendrite growth and corrosion that took place on Zn anodes in rechargeable aqueous batteries with different additives of organic molecules, including cetyltrimethylammonium bromide (CTAB), sodium dodecyl sulphate (SDS), polyethylene-glycol (PEG-8000) and thiourea (TU). The authors evaluated the battery performance based on the surface properties of its Zn electrode and found that the surface texture and other properties of the Zn anode displayed higher resistance with dendrite formation, with a reduction in corrosion-current density of up to eightfold as analysed by a Tafel test. Based on the XRD, the effects of organic additives on the crystallographic orientation and the surface morphology indicated different orientation of zinc crystal growth, and the zinc electrode with the SDS additive had the highest corrosion-free and dendrite-free properties among other additives as demonstrated in Fig. 3d. In addition, the organic additives had a significant impact on the zinc plating morphology as shown in SEM images (Fig. 3e). The morphology of Zn-CTAB was porous needle-like crystals with uniform size distributed evenly, which affected the electron transfer kinetics and increased the competition between nucleation and crystal growth of the surface. In contrast, the morphologies of the Zn-SDS and Zn-PEG were smooth and uniformly distributed, with grown perpendicular to the substrate and in various directions. The active site and nucleation rate were reduced as additives absorbed onto a substrate surface as claimed by the authors. Similarly, to Zn-SDS and Zn-PEG, Zn-TU deposits were uniform and regular but not compact. This was attributed to the presence of hydrogen gas bubbles blocked some of the spots on the electrode surface. The SEM image of Zn–No showed an irregular layer structure with some features growing perpendicularly to the surface, which may affect the separator of the battery. The surface of the commercialised zinc was flat and smooth with some defects/holes that might be existed due to manufacturing issues. Moreover, the electrodes prepared with electrolytes in contact with the above-mentioned additives showed up to a 30-fold more corrosion-free surface compared to electrodes without any additives. Also, higher capacity retention rates of 79%, 76% and 80% were obtained with Zn-SDS, Zn-PEG and Zn-TU anodes, respectively, after a long cycle life (1000 charge–discharge cycles) at a 4C rate, where (1C = 120 mA g^−1^) as shown in Fig. 3f. Nevertheless, commercial zinc foil (without additives) displayed a capacity retention of only 67%, while the capacity suddenly dropped when Zn-CTAB batteries were run 350 times due to dendrites formation, as claimed by the authors. When CTAB surfactant was strongly adsorbed onto the anode surface, the active area might be strongly blocked leading to lower kinetics of the electron transfer and negatively affecting the surface properties of the Zn anode. Other features were exhibited with the Zn anodes that incorporated additive-containing electrolytes, such as lower open-circuit potential and smaller float-charge current. However, these additives were examined with two types of batteries including coin cells and two-electrode Swagelok^TH^, while the Zn-based redox flow batteries (e.g. zinc–bromine flow batteries) were neglected. Thus, different types of flow batteries should be taken into consideration to further examine the effects of these organic additives and their mechanisms when using metallic zinc anodes.

**Fig. 4:**
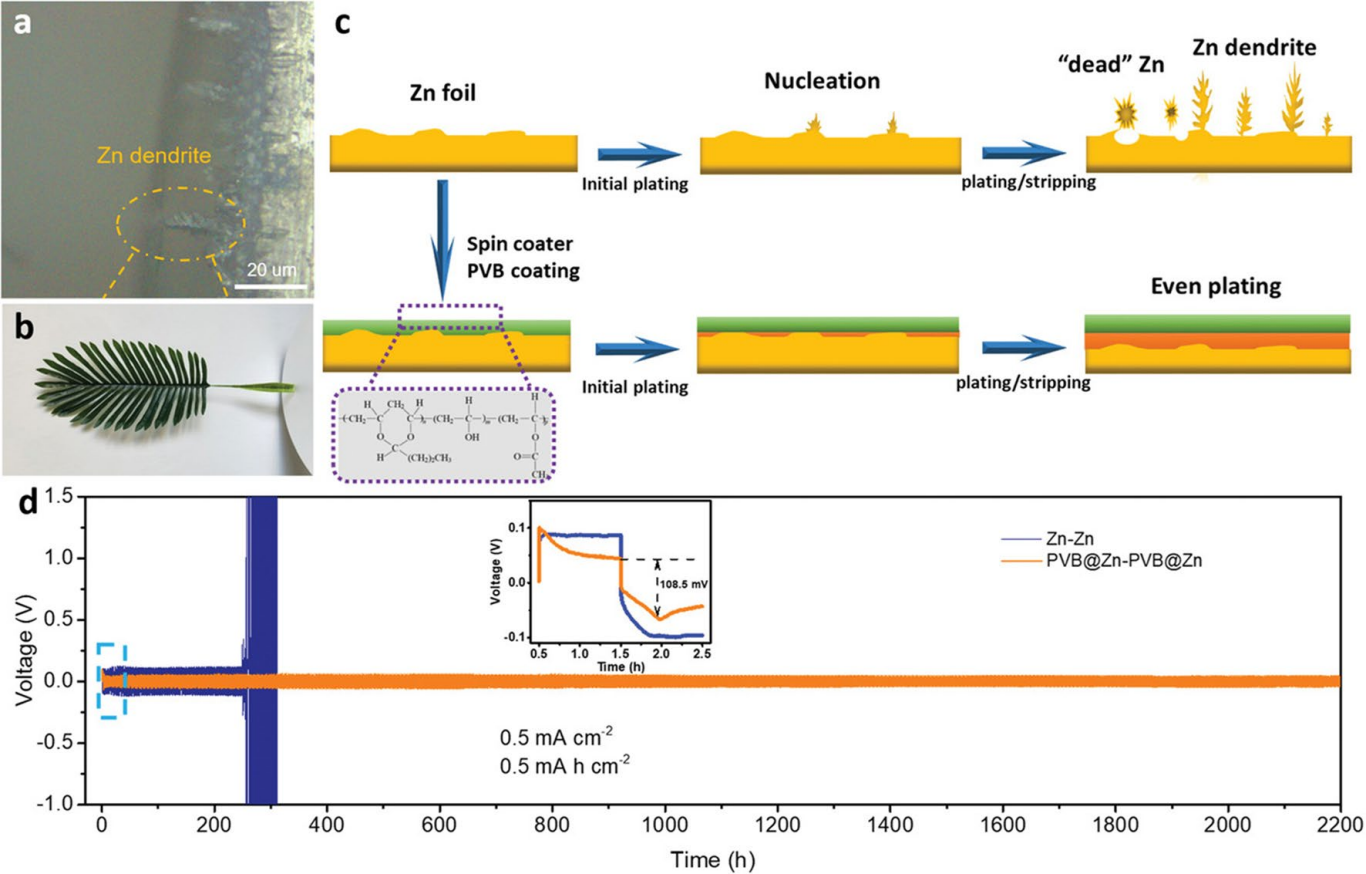
Zn dendrite morphology and schematic illustration of Zn plating/stripping: **a** Optical microscope image of Zn dendrites on a cross section of Zn foil. **b** Photograph of a palm leaf, similar to the morphology of Zn dendrites. **c** Schematic illustration of morphology evolution for both the bare Zn–Zn cell and the PVB@Zn-PVB@Zn cell during repeated cycles of stripping/plating. **d** Cycling stability of Zn plating/stripping in both bare Zn and PVB@Zn symmetric cells, with the inset showing the initial voltage profiles of both cells. Panels **a**–**d** reproduced with permission from Ref. [83]. Copyright 2020, WILEY-VCH Verlag GmbH & Co. KGaA, Weinheim

Furthermore, Hao et al. [83] investigated the surface chemistry of zinc in slightly acidic electrolytes (ZnSO_4_) and the effect of dendrite growth on the electrochemical performance of Zn-based batteries. They revealed a poor Zn metal performance of the bare electrode in the mild electrolyte due to porous by-product (Zn_4_SO_4_(OH)_6_·*x*H_2_O) formation and dendrite growth. The initial understanding of the dendritic problem and side reactions guided them to effective suppression solution achieved via an artificial solid/electrolyte interphase (SEI) layer produced by a polymer film of poly(vinyl butyral) (PVB) that was applied to the Zn anode using a simple spin-coating process. This insulating polymer layer displayed excellent hydrophilicity and ionic conductivity due to the abundant polar groups in PVB chains, inhibiting the side reactions and Zn dendrite growth as revealed in Fig. 4a–c. In the symmetrical Zn cell, the side-reaction-free, dendrite-free PVB@Zn anode permitted repeated plating/stripping process over 2200 h (Fig. 4d), a much greater period than for bare Zn cells. When coupled with PVB@Zn anodes, MnO_2_ battery systems exhibited higher CE and longer lifespans compared to batteries using bare Zn anodes. However, more studies are required to investigate the effect and stability of PVB@Zn anodes if this strategy is adopted in zinc–bromine flow batteries. In addition, creating future zinc–bromine flow battery should be focused on employing multifunctional components that will maximise the system's utility while reducing manufacturing and maintenance costs.

**Fig. 5:**
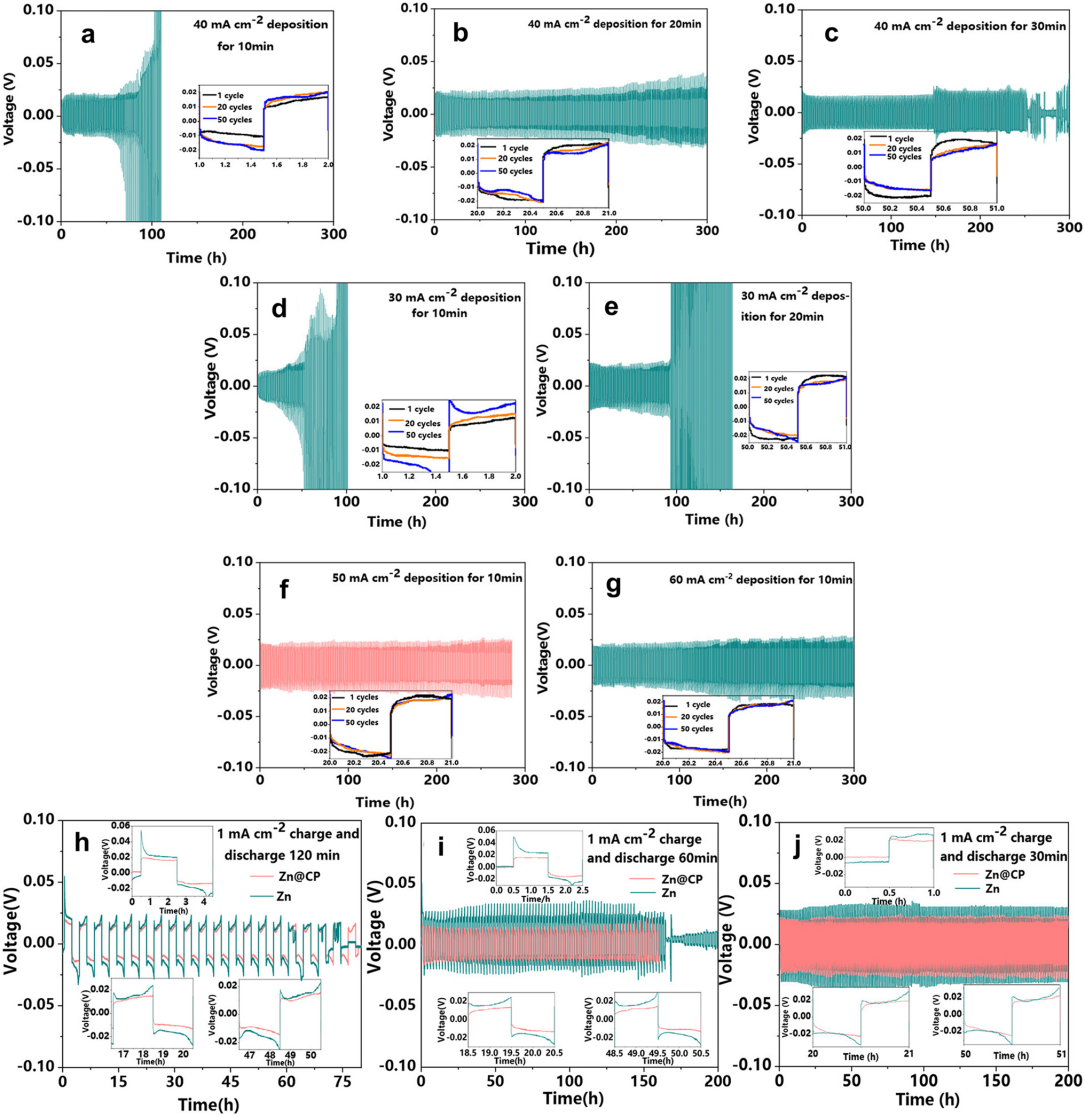
Polarisation test of Zn@CP electrode prepared via electrodeposition under various conditions: **a** 40 mA cm^−2^ deposition for 10 min, **b** 40 mA cm^−2^ deposition for 20 min, **c** 40 mA cm^−2^ deposition for 30 min, **d** 30 mA cm^−2^ deposition for 10 min, **e** 30 mA cm^−2^ deposition for 20 min, **f** 50 mA cm^−2^ deposition for 10 min, and **g** 60 mA cm^−2^ deposition for 10 min. Electrochemical performance of Zn@CP-50 and Zn plate electrodes under 1 mA cm^−2^ for various time durations: **h** 120 min, **i** 60 min, and **j** 30 min. Panels **e**–**n** reproduced with permission from Ref. [107]. Copyright 2020, American Chemical Society

In another study, Wu et al. [107] developed anodes for zinc-ion batteries that were made from zinc electrodeposited on carbon paper (Zn@CP). The authors observed that the morphology and electrochemical performance of Zn@CP were strongly influenced by the deposition time and electrodeposition current density during preparation (Fig. 5a–e), and this might be due to nucleation activation energy before electrodeposition. The electrode prepared with Zn@CP under 50 mA cm^−2^ and 10-min deposition time exhibited superior electrochemical performance, with a cycle life of 280 h and a small hysteresis of 22 mV as shown in Fig. 5f, compared to the Zn@CP electrodes (Fig. 5a–g), indicating the crucial impact of the preparation current density on the electrochemical performance. Therefore, Zn@CP-50 electrode was selected for further study, and the Zn plate was used as a control sample. Less polarisation was observed with Zn@CP-50 electrode compared to the Zn plate in symmetric cells that were cycled at different times (30, 60 and 120 min) in each cycle, under a constant current density of 1 mA cm^−2^ (Fig. 5h–j). The results of this study can contribute to the design of Zn-based composite anode materials for zinc–bromine flow batteries to achieve long-term operation with high performance. In addition, electrochemical properties of the Zn electrode can be enhanced by the design of the zinc/carbon (Zn/C) composite electrodes [108]. A fast-electron transport channel was achieved by covering the Zn electrode with carbon nanotubes (CNT), which allowed a uniform zinc deposition. However, all the above-mentioned solutions have not been thoroughly examined and there is a big opportunity to use and further assess these applications in the context of zinc–bromine flow batteries.

**Fig. 6:**
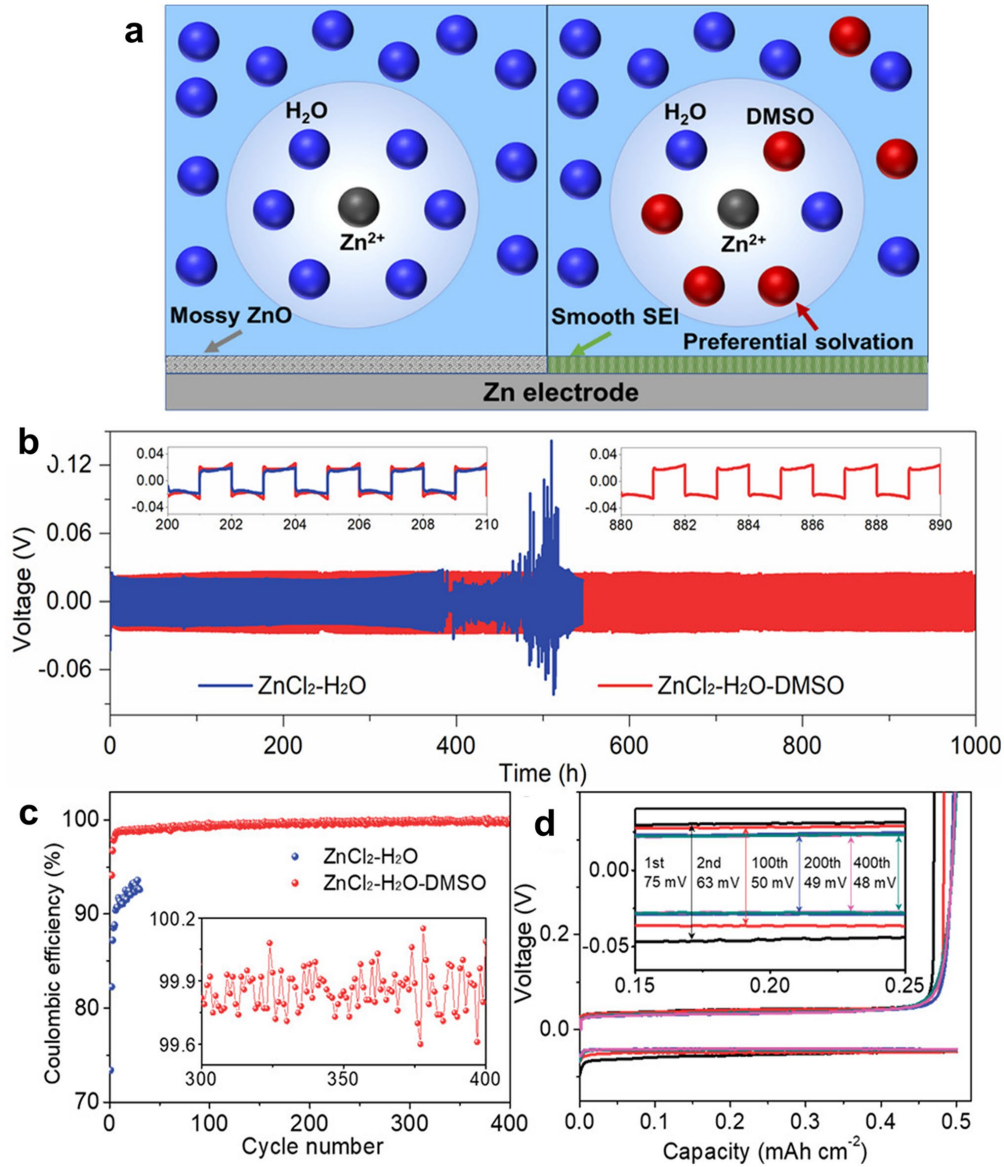
**a** Schema of Zn^2+^ solvation structure and zinc surface passivation in H_2_O (left) and H_2_O–DMSO (right) solvents Galvanostatic. **b** Zn plating/stripping in Zn||Zn symmetrical cells at a current density of 0.5 mA cm^−2^ and a capacity of 0.5 mAh cm^−2^, **c** Zn plating/stripping CE in different electrolytes at 1 mA cm^−2^ and 0.5 mAh cm^−2^, and **d** voltage profiles of Zn plating/stripping processes at selected cycles in ZnCl_2_–H_2_O–dimethyl sulphoxide (DMSO) electrolytes. Panels **a**–**d** reproduced with permission from Ref. [109]. Copyright 2020, American Chemical Society

As ZBRB uses an aqueous solution of different primary and supporting salts and a bromine complexing agent (BCA). During the charge/discharge process, the electrochemical potential of this system (1.8 V) is higher than the hydrolysis potential of water (1.23 V), which leads to the hydrogen evolution reaction (HER) and the formation of different oxidised states that can alter/degrade the electrodes and decompose organic components such as the BCAs. HER is a serious challenge produced by ZBRBs during charging due to the reaction between zinc and water, which reduces CE, consumes water and facilitates zinc dendrite growth [14]. In general, there are six water molecules surrounding Zn^2+^ cations in an aqueous solution, resulting in a bond length of 208–210 pm between the Zn^2+^ and H_2_O molecules and an overall diameter of 416–420 pm between them. The strong coulombic interactions between solvated Zn^2+^ ions and their surrounding H_2_O shell accelerate parasitic water reduction during charging, where zinc is deposited on the anode surface [109]. Zn^2+^-insulating passivates (such as zinc hydroxides) are formed on the zinc surface when the pH of the local environment increases due to H_2_ evolution [109]. This decreases zinc activity and cycle life and allows zinc dendrites to grow. Different additives can be used to suppress water reduction and protect zinc electrodes. Cao et al. [109] studied the addition of DMSO into the ZnCl_2_–H_2_O electrolyte in aqueous zinc batteries to weaken the bonding strength between the Zn^2+^ ions and solvated H_2_O (Fig. 6a). In this study, a symmetrical Zn||Zn cell with a ZnCl_2_–H_2_O electrolyte demonstrated stable cycling behaviour for only 390 h with a ~ 20.0 mV overpotential. Thereafter, the polarisation voltage rose suddenly and irreversibly, and the cell failed due to zinc dendrite growth. A stable polarisation overpotential of ~ 20.5 mV and extended cycling life of 1000 h was obtained with the Zn||Zn cell in ZnCl_2_–H_2_O–DMSO, which represents a 2.5-fold improvement in cycle life (Fig. 6b–d). The authors of this study argued that a uniform SEI containing Zn-ion conductive materials should be constructed, allowing for Zn^2+^ transport while preventing H_2_O from permeating the surface of the zinc anode. Zhang et al. [110] investigated the feasibility of the LiCl–ZnCl_2_ (water-in-salt)-mixture-concentrated electrolyte on the hydrogen bonding interruption of water molecules and found that Zn^2+^ ions can coordinate with Cl^−^ rather than H_2_O, leading to strong O–H covalent bonds while decreasing the solvation activity of H_2_O in the electrolyte. These approaches are worth adapting to ZBFBs to mitigate Zn/H_2_O reactivity and suppress the decomposition of solvated H_2_O.

To this end, it is essential to determine suitable solutions for better activities of zinc electrodes, assess their suitability for the system and compare them to alternatives using different approaches that could improve the performance of ZBRB systems.

### Bromine Half-Cell

Bromide ions (Br^−^) release electrons and oxidise to bromine (Br_2_) at the positive electrode of ZBRBs during charge, while the reversible reactions occur in the following discharge process. The electrochemical reactions of bromide oxidation/reduction on the positive electrode are determined by Eqs. 3 and 4:

3 $$2{\text{Br}}^{ - } \to {\text{Br}}_{2} + 2e^{-} \;({\text{Charging}})$$

4 $${\text{Br}}_{2} + 2e^{ - } \to 2{\text{Br}}^{ - } \;({\text{Discharging}}) \;(E^{^\circ } = 1.087\;{\text{vs}}{\text{. SHE at}}\;25\,{^\circ } {\text{C}})$$

Great attention has been given to Br_2_/Br^−^ redox couple reactions to investigate the mechanisms and kinetics of bromine in ZBRBs. From the 1960s onwards, bromine oxidation and reduction reactions have been studied on a platinum electrode [111–113]. Three mechanisms have been proposed for bromine–electrode reactions: (1) Volmer–Heyrovsky (V–H), (2) Volmer–Tafel (V–T) and (3) Heyrovsky–Tafel (H–T) mechanisms [114].

The anodic process produces molecular bromine by oxidising Br^−^ ions, while the reverse reaction occurs in the cathodic process, and several steps are involved in the reaction. A Volmer reaction is conducted in the anodic process to create adsorbed bromine atoms by discharging Br^−^ ions [115].

5 $${\text{Br}}^{-} + {\text{Br}}_{{({\text{ad}})}} \leftrightarrow {\text{Br}}_{2} + e^{-} \;({\text{Volmer reaction}})$$

The formation of molecular bromine can then be achieved using one of the two following processes: the discharge of Br^−^ ions on adsorbed bromine atoms or a combination of bromine atoms adsorbed on the surface as expressed by Eqs. 6 and 7:

6 $${\text{Br}}^{-} {\text{ + Br}}_{{({\text{ad}})}} \leftrightarrow {\text{Br}}_{2} + e^{-} \;({\text{Heyrovsky reaction}})$$

7 $$2{\text{Br}}_{{({\text{ad}})}} \leftrightarrow {\text{Br}}_{2} \;({\text{Tafel reaction}})$$

In general, it has been demonstrated that either the V–H mechanism or the V–T mechanism, with the Volmer reaction controlling, is an acceptable mechanism for the Br_2_/Br^−^ reaction according to the qualitative comparison of the outcomes of the models, which is useful in predicting current density–overpotential curves [114, 115].

The bromine reaction mechanism on carbon materials has also been identified. Janssen and Hoogland [116] investigated the electrochemical mechanism of bromine at a graphite electrode surface. They found that bromine is produced based on the V–H mechanism, where the Heyrovsky step determines the reaction rate. Another study elucidated the Br_2_/Br^−^ redox reaction with two different vitreous carbon electrode materials, including reticulated vitreous carbon and smooth vitreous carbon, using the rotating disc electrode technique [117]. Both electrodes undergo two consecutive electrochemical steps: the Volmer and Heyrovsky reactions. In the cathodic process, the Volmer reaction was the rate-controlling step of the adsorbed Br^−^ ion at the electrode surface, while the Heyrovsky reaction was the rate-determining step in the anodic process where Br^−^ ion is combined with the Br atom [117]. When Br_2_ is produced, Br_3_^−^ is formed in a rapid equilibrium, and then a reduction of Br_2_ occurs based on V–H mechanisms. Elemental bromine mainly reacts with bromide ions in the aqueous electrolyte producing different polybromide ions [118]. According to Eq. 8, tribromide ions (Br_3_^−^) initially form and occur close to the electrode surface. This followed by the formation of pentabromide (Br_5_^−^) and finally heptabromide (Br_7_^−^) that take place in the solution [115].

8 $$n{\text{Br}}_{2} + {\text{Br}}^{-} \leftrightarrow {\text{Br}}_{2n + 1}^{ - } \;(n = 1, 2, 3)$$

The aqueous ZBRB is the best representative example of the halogen-based rechargeable batteries as the heavy metal Br_2_ redox couple possesses higher theoretical specific capacities (335 mAh g^−1^) compared to that of I_2_ redox couple (211 mAh g^−1^) [119]. In addition, the redox kinetics of bromine are fast and reversible, making it a particularly good electroactive species. However, it has a potential of about 1.07 V that is close to the oxygen evolution potential in neutral electrolytes. This prevents 100% full oxidation of Br^−^ ions during the charging process as the oxygen evolution consumes some of the electrons provided to the bromine cathode, limiting its performance. While the high solubility of bromine and its derivatives (e.g. Br_3_^−^) enhances their redox kinetics in the system, bromine can easily diffuse through the membrane to the zinc side causing severe self-discharge. Further, bromine is thermodynamically corrosive to zinc and other parts in the battery system (e.g. pipes), which may eventually lead to cell failure over long cycle times. Besides, the nature of Br_2_ is volatile and hazardous to health and may escape into the external environment as a gas. Hence, it should be stabilised with complexing agents that reduce its reactivity and vapour pressure without affecting its electrochemical properties [120]. Incorporating quaternary ammonium salts into the electrolyte minimises the magnitude of this problem [113, 120]. Different types of quaternary complexes (also known as bromine sequestering agents) are typically added to the static and flow ZBRB electrolytes to sequester bromine into an oily phase with low vapour pressure, producing a non-aqueous polybromide complex that sinks to the bottom of the catholyte tank via gravitational action [121, 122]. In addition to the complexing agents, anti-corrosive materials can be used for the cell components (e.g. pipes) to reduce maintenance and extend the battery’s life. However, the complex state of Br_2_ limits the kinetics of the Br_2_/Br^−^ redox reaction and affects the battery’s performance.

**Fig. 7:**
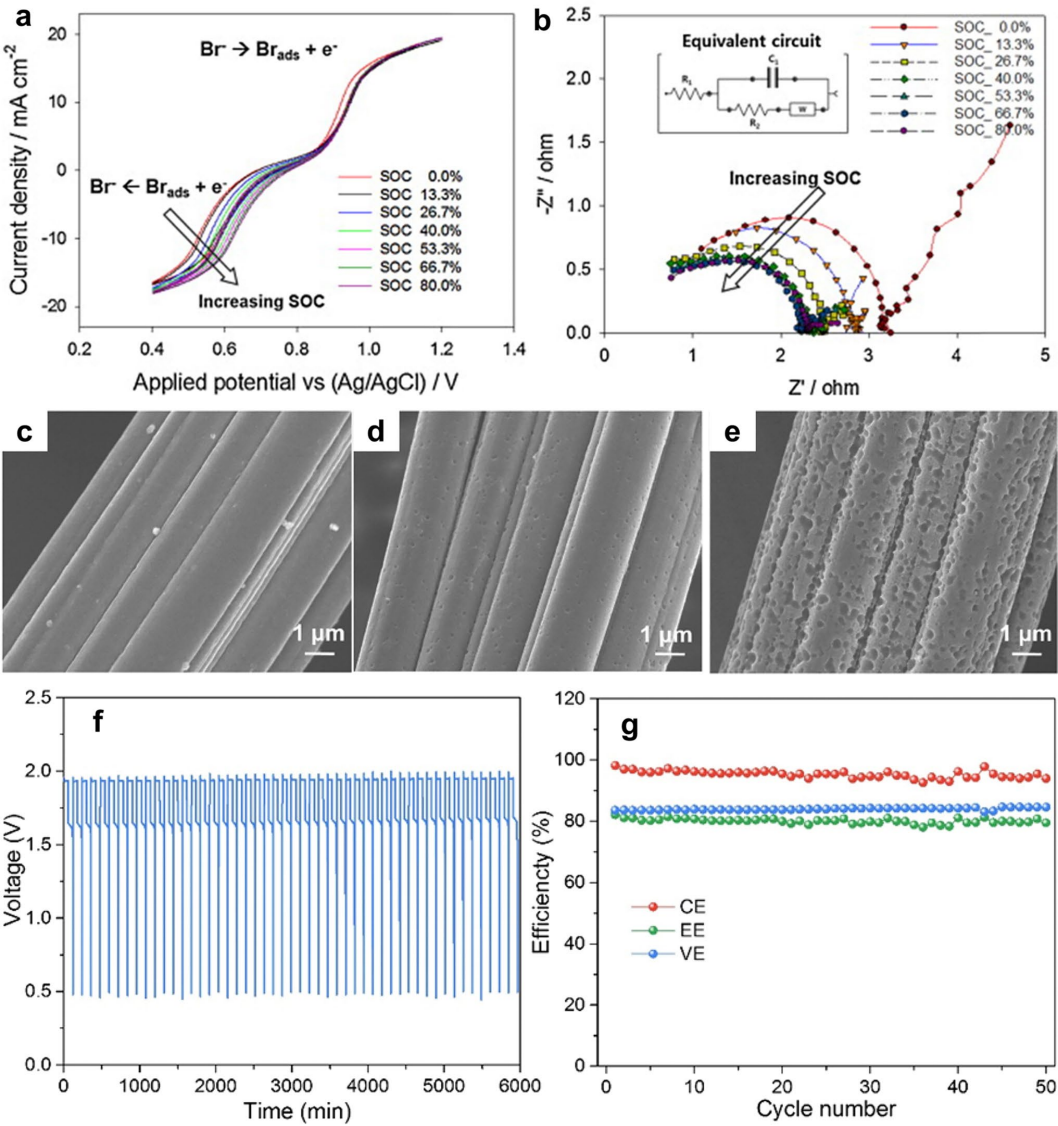
Electrochemical characteristics of the catholyte at various SoCs for the first cycle of charge–discharge test: **a** Cyclic voltammetry (the potential scan started at 0.85 V forward and then backward with a scan rate of 500 mV s^−1^) and **b** electrochemical impedance spectroscopy analysis (measured under the frequency range of 10 kHz–10 mHz with potential perturbations of amplitude 10 mV). Panels **a** and **b** reproduced with permission from Ref. [124]. Copyright 2014, Elsevier Ltd. SEM images of **c** pristine GF, **d** GF-2 h and **e** GF-4 h, and cycling performance of a ZBFB with GF-2 h electrode. **f** Voltage versus time plot and **g** Columbic, voltage and energy efficiencies during the 50 charge–discharge cycles. Panels **c**–**g** reproduced with permission from Ref [125]. Copyright 2017, Elsevier B.V

The most common organic BCAs used to capture bromine in ZBFBs are methyl-ethyl-morpholinium bromide and/or methyl-ethyl-pyrrolidinium bromide [123]. Many studies have investigated the effect of BCAs on bromine kinetics reactions. Jeon et al. [124] revealed the impact of BCAs on the charge transfer reaction of a bromine electrode in ZBFB using in situ electrochemical characterisation and impedance analysis under various states-of-charge (SoCs), as shown in Fig. 7a, b. The authors found that the charge transfer resistance for Br_2_/Br^−^ oxidation significantly decreased with an increase in the SoC, where the adsorption of bromide ions was positively affected by accumulated polybromide complex on the Br-side electrode surface. They also argued that polybromide with an amphiphilic property can permit interconnection between hydrophobic electrode surfaces and hydrophilic bromide ions.

Bromine formation, sequestration and their reverse processes are expected to occur at a lower rate than the zinc-side reactions [126]. In more details, the bromine-side electrochemical kinetics have slower rate in comparison to the zinc-side electrochemical kinetics, and thus affect battery performance significantly. This is because that interactions with the BSA cause additional mechanistic steps (namely binding or dissociation, depending on the charging or discharging of the ZBB, respectively), which in turn cause mass transport limitations in the system [68]. Therefore, a higher active surface area is required for the bromine-side electrode to prevent a reduction in the exchange current density owing to the slow reaction rate. A specific catalytic centre, such as O functional groups or N centres on the electrode surface, may also enhance redox reaction kinetics [123]. In addition, the highly corrosive nature of bromine requires corrosion-resistant and durable electrode materials, and positive electrodes require good electrochemical activity and reversibility of Br_2_/Br^−^ redox reactions [115]. Consequently, as ZBFB became industrially viable, carbon-based electrodes became more prevalent. Furthermore, electrochemical studies have been carried out using graphite, vitreous carbon, carbon felt and carbon–plastic composite electrodes due to their higher charge transfer capability [26, 67, 117, 127]. Carbon materials are excellent choices for bromine electrodes in ZBRBs since they are cheaper and have excellent electrical conductivity and chemical resistance, a wide operating range and modifiable surface properties [115]. In addition, the high surface area and pore size distribution of carbon materials are the key features affecting the Br_2_/Br^−^ electrochemical redox activity in ZBFBs. The high surface area of a carbon layer (> 1500 cm^2^ g^−1^) is necessary for the positive electrode to promote the slow kinetics for Br_2_/Br^−^ caused by the complicated chemistry of bromide ions in aqueous solutions, including (1) generation of ZnBr_*n*_^2−*n*^ complexes, (2) charge transfer reaction, (3) polybromide anion complexation and (4) Br_2_ sequestration by BCAs [63]. Wang et al. [128] investigated the performance of different structures of four commercialised carbons, including acetylene black (AB), expanded graphite (EG), carbon nanotube (CNT) and BP2000 (BP), as well as their effect on the activity of Br_2_/Br^−^ couples in ZBFB. The authors stated that the BP showed the highest activity among the carbon materials due to its high specific surface area, which provides more active sites for the electrochemical reaction of Br_2_/Br^−^. However, corrosion is more likely to occur with BP under a bromine redox reaction in ZBFBs. Wang et al. [36] reported a cage-like porous carbon material with exceptionally high activity on Br_2_/Br^−^ and the entrapping capability of Br_2_ complex, which could effectively promote electrode activity and suppress Br_2_ crossover in a bromine-based flow battery. Wu et al. [125] thermally treated GF electrodes at 500 °C for different durations to improve the electrocatalytic activities of GF and thus enhance the electrochemical activity of the Br_2_/Br^−^ redox reaction in ZBFB. The authors argued that the surface of pristine GF fibres was smooth with some ravines before thermal treatment (Fig. 7c). A number of pores were formed on the fibre’s surface after being treated thermally at 500 °C for 2 h (Fig. 7d). Meanwhile, a large number of pores were generated and collapsed together to form larger pores, and the treatment duration was increased to 4 h (Fig. 7e). The efficiency also increased, reaching up to 81.8% at a current density of 40 mA cm^−2^, with good stability during cycling tests and a relatively stable charge voltage in addition to a slight increase in the discharge voltage, leading to an overall improvement in voltage efficiency, as shown in Fig. 7f, g. While some progress has been made, additional work is needed to further improve the bromine electrode performance in ZBRBs.

### Other Fundamental Aspects in Zinc–Bromine Rechargeable Batteries

**Fig. 8:**
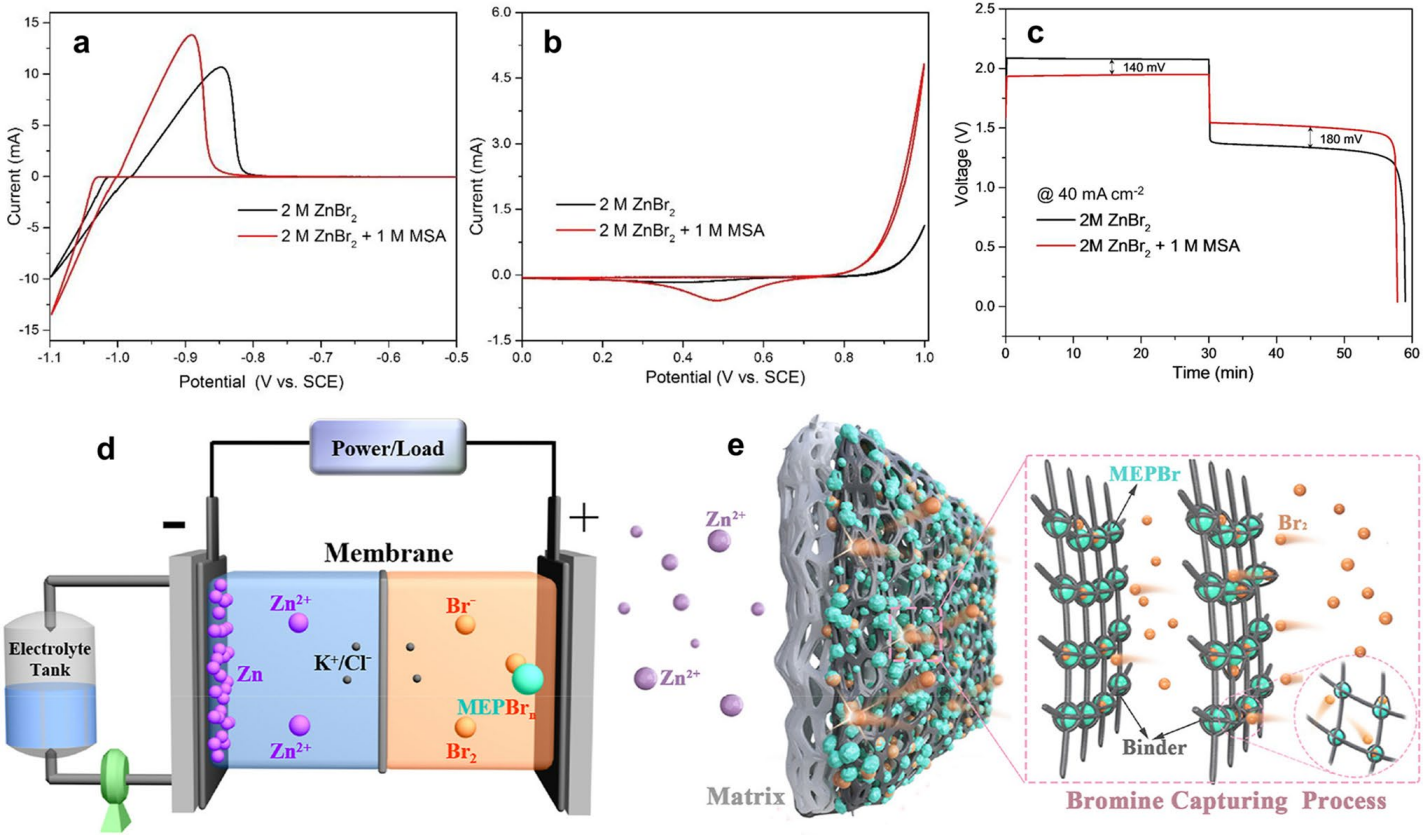
CV results of **a** Zn^2+^/Zn, and **b** Br_2_/Br^−^ redox reaction at a scan rate of 20 mV s^−1^ in 2 M ZnBr_2_ with and without 1 M MSA. **c** Charge–discharge profiles obtained at a current density of 40 mA cm^−2^. Panels **a**–**c** reproduced with permission from Ref. [129]. Copyright 2018, Elsevier B.V. **d** Schematic diagram of a ZBSFB and **e** the used highly selective porous composite membrane with bromine capturing capacity. Panels **d** and **e** reproduced with permission from Ref. [130]. Copyright 2021, Elsevier Ltd

An aqueous solution of ZnBr_2_ is used as the electrolyte in ZBRBs. It consists of zinc–bromide as a primary electrolyte (1–4 M), which participates in charge transfer reactions with the electrodes to facilitate the storage of energy within the system [68]. However, in practice, supporting secondary salts (e.g. ZnCl_2_ and KCl) are normally used to promote ionic conductivity and lower internal resistance due to the low conductivity of zinc–bromide solution, thereby increasing the battery’s energy efficiency [109]. Wu et al. [129] employed methane sulphonic acid (MSA) as a supporting electrolyte to improve the conductivity and mitigate zinc dendrite in ZBFB. The authors revealed an improvement in the kinetics and reversibility of the redox couples of both battery sides (Fig. 8a, b) after modifying the electrolyte with 1 M MSA. Moreover, the internal resistance was clearly reduced from 4.9 to 2.0 Ω cm^2^, leading to better charge voltage plateau (Fig. 8c) and higher energy efficiency from 64 to 75% at a current density of 40 mA cm^−2^. While MSA could be a promising supporting electrolyte for ZBFBs, it is necessary to conduct various experiments under different conditions to ensure its reliability.

In addition to the supporting salts, other fundamental aspects play a crucial role in battery performance based on electrolyte composition. Specifically, the pH and reactant concentrations of the electrolyte have a critical impact on battery performance during charge. A pH that is too high will produce zinc hydroxide precipitation with a moss-like appearance of zinc deposits in weakly acidic and basic electrolytes, while a pH that is too low will cause excessive corrosion of zinc [131]. In addition, the significant variation in the reactant concentrations reflects the charge/discharge cycle stage [68]. The concentration decreases as the battery charges and Zn^2+^ is plated out and Br^−^ is oxidised to Br_2_ before climbing back up as the battery discharges and recovers its original ZnBr_2_ concentration [68]. Both the reactant concentration alteration and the ion migration between circuits could affect the electrolyte pH and electrode overpotential. As such, electrolyte composition should be considered and improved for higher performance and longer operational life of the battery system.

The membrane is another integral component of ZBFBs that prevents cross-contamination, especially elemental bromine and bromide ions, and reduces the possibility of electrical contact between electrodes. The selection of an appropriate membrane can improve the performance of both zinc anode and bromine cathode simultaneously. Important characteristics that a suitable membrane should possess include high ionic exchange capacity, low internal resistance and low cost [67, 132, 133]. In addition, it is necessary to have high selectivity to distinguish between bromide anions and zinc cations that should be allowed to pass through the membrane. Hua et al. [130] designed a highly selective porous composite membrane with a highly selective separation layer for bromine-based flow batteries as presented in Fig. 8d, e, respectively. This study proposed that diffused bromine and complexing agents in the non-porous selective layer of the membrane could be complexed. Therefore, bromine diffusion could be captured by the separation layer of the porous composite membrane, thus preventing the battery from self-discharge. For electrochemical reasons, whenever it is possible, it is critical to minimise the diffusion of Br_2_ to the electroplated zinc, which can be consequently oxidised during charging, causing self-discharge and lower CE for the system [68].

A high structural resistance membrane is also crucial to prevent possible short-circuit due to piercing by large zinc dendrites, ensuring long-term battery safety and reliability [134]. In terms of morphology, membranes can be generally classified into porous and ion-exchange membranes (e.g. Nafion®), which are both appropriate and capable of separating the anode and cathode electrolytes in ZBFBs. Porous membranes are defined as macroporous (> 50 nm), mesoporous (2–50 nm) or microporous (0.2–2 nm) depending on their average pore diameter, while non-porous membranes transport ions via solution–diffusion mechanisms [134].

**Fig. 9:**
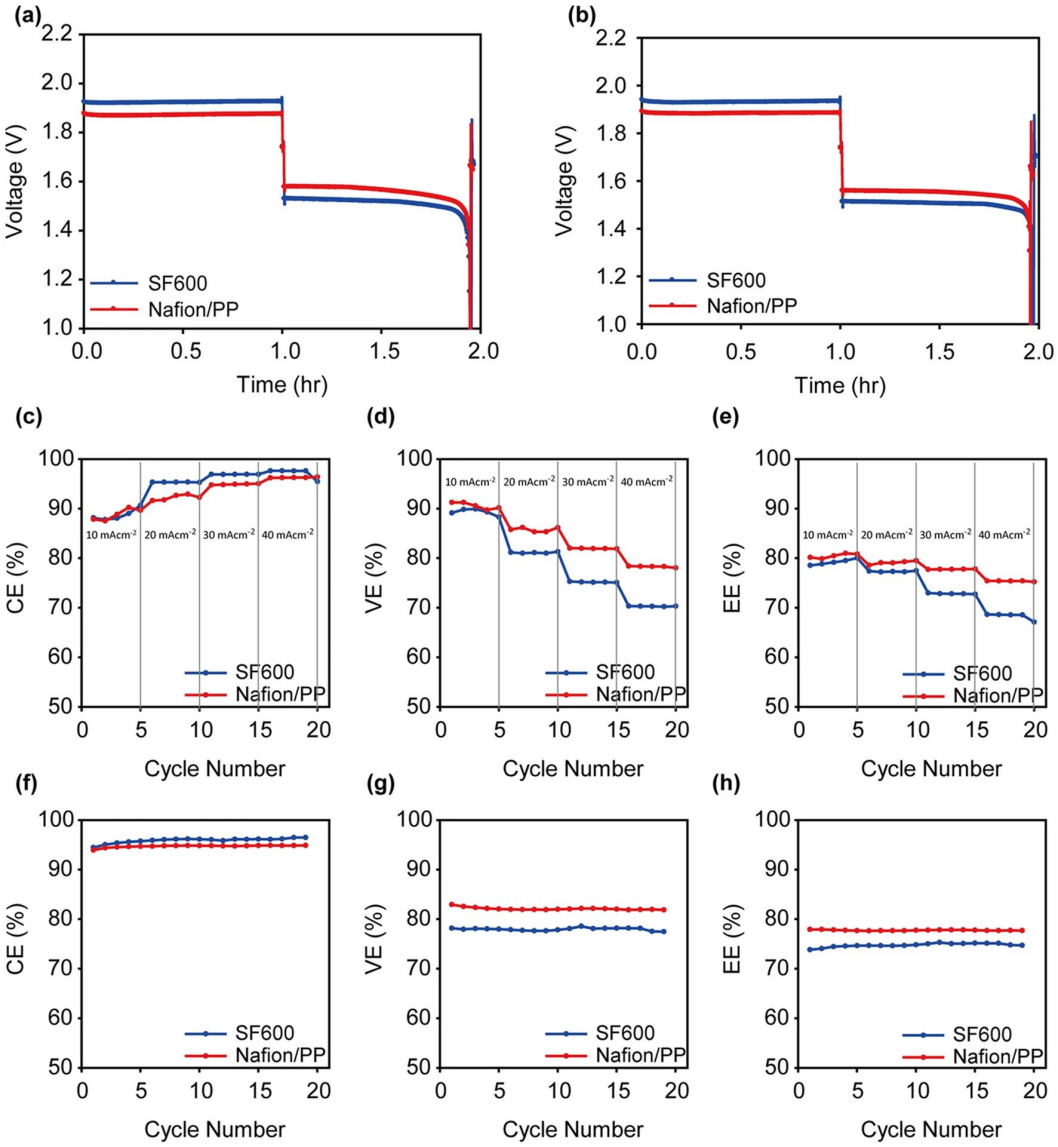
The charge–discharge curves of the Nafion/PP and SF-600-based ZBB single cells at **a** the first cycle and **b** the 19th cycle. **c** Coulombic, **d** voltage, and **e** energy efficiencies of the Nafion/PP and SF-600-based ZBB single cells with various current densities from 10 to 40 mA cm^−2^. **f** Coulombic, **g** voltage, and **h** energy efficiencies of the Nafion/PP and SF-600-based ZBB single cells with cycling at 20 mA cm^−2^. Panels **a**–**h** reproduced with permission from Ref. [135]. Copyright 2017, Elsevier Ltd

Various membrane types have been explored, including cation or anion-exchange membranes, composite membranes and woven nanofiber membranes [134], each of which has different advantages and disadvantages, with these factors altering the performance of ZBFBs. Furthermore, hydrophilic-treated porous polyethylene membranes, such as SF-600 (Asahi Kasei) and Daramic®, have been investigated in relation to the balance between ionic conduction and Br_2_ crossover. While these membranes are relatively thick and therefore have increased resistance, they have been shown to effectively prevent Br_2_ crossover through the pores [135]. In contrast, non-porous Nafion membranes can also be used for ZBFBs as they can effectively block bromine migration. A void-free Nafion/polypropylene (Nafion/PP) membrane in ZBFBs has been successfully fabricated in a previous study [135]. In this work, smaller area-specific and far lower Br_2_ diffusivity was achieved via the designed ultrathin Nafion-filled porous membranes compared to SF-600 porous membranes due to their dense morphology, albeit that they were 37.5 times thinner. It was clearly observed that the charge–discharge voltage profiles (Fig. 9a, b) were reduced and highly stable at two selected cycles (1st and 19th cycle) indicating less polarisation for the cells with Nafion/PP relative to that with SF-600 membranes. The rate capability of ZBRB cells investigated with Nafion/PP at different current densities (Fig. 9c–e) outperformed cells with SF-600 membrane. While CE of the Nafion/PP and SF-600-based ZBRBs was clearly equivalent, the voltage and energy efficiencies of cells with Nafion/PP were higher (Fig. 9f–h), demonstrating efficient approach for better battery performance. As this study indicated that the modification of Nafion membrane could achieve better outcomes such as high quality with low cost, more research is essential to investigate various membrane qualities for better ZBRB performance.

## Assessment Methods and Performance Metrics

The research community has employed many diagnostic techniques to examine virtually every aspect of zinc–bromine batteries. Electrochemical, physical and chemical phenomena are studied to determine material properties, operational losses, and transport and integrated system properties, which, in turn, influence efficiency, durability, power and capacity. Testing methods and performance metrics vary, making it difficult to compare the reported zinc-based batteries and evaluate their potential for practical application. This section discusses various assessment methods and metrics that relate directly to the working principles and degradation mechanisms of ZBRB (static and flow) chemistries to facilitate their practical development and future benchmarks.

There have been extraordinary advances in battery characterisation techniques with the development of sophisticated diagnostic tools (e.g. nuclear magnetic resonance, X-ray diffraction, optical microscopy, transmission electron microscopy), which can ideally be used in situ as a battery is cycled. Despite this, more work is needed to push the boundaries of existing methods and to design operating analytical techniques (for use under real conditions) for monitoring battery health and extending battery life, as well as for the design of new materials and the optimisation of existing and new electrochemistry technologies. In the literature, operando and in situ are almost interchangeable terms referring to measurements on a system under real working conditions [136]. Typically, an operando measurement is a measurement taken while a battery is operating (cycling). An in situ measurement (meaning on site) consists of measuring a variable against a parameter relevant to the system, such as time, temperature, pressure or other variables, to minimise its degradation [136].

**Fig. 10:**
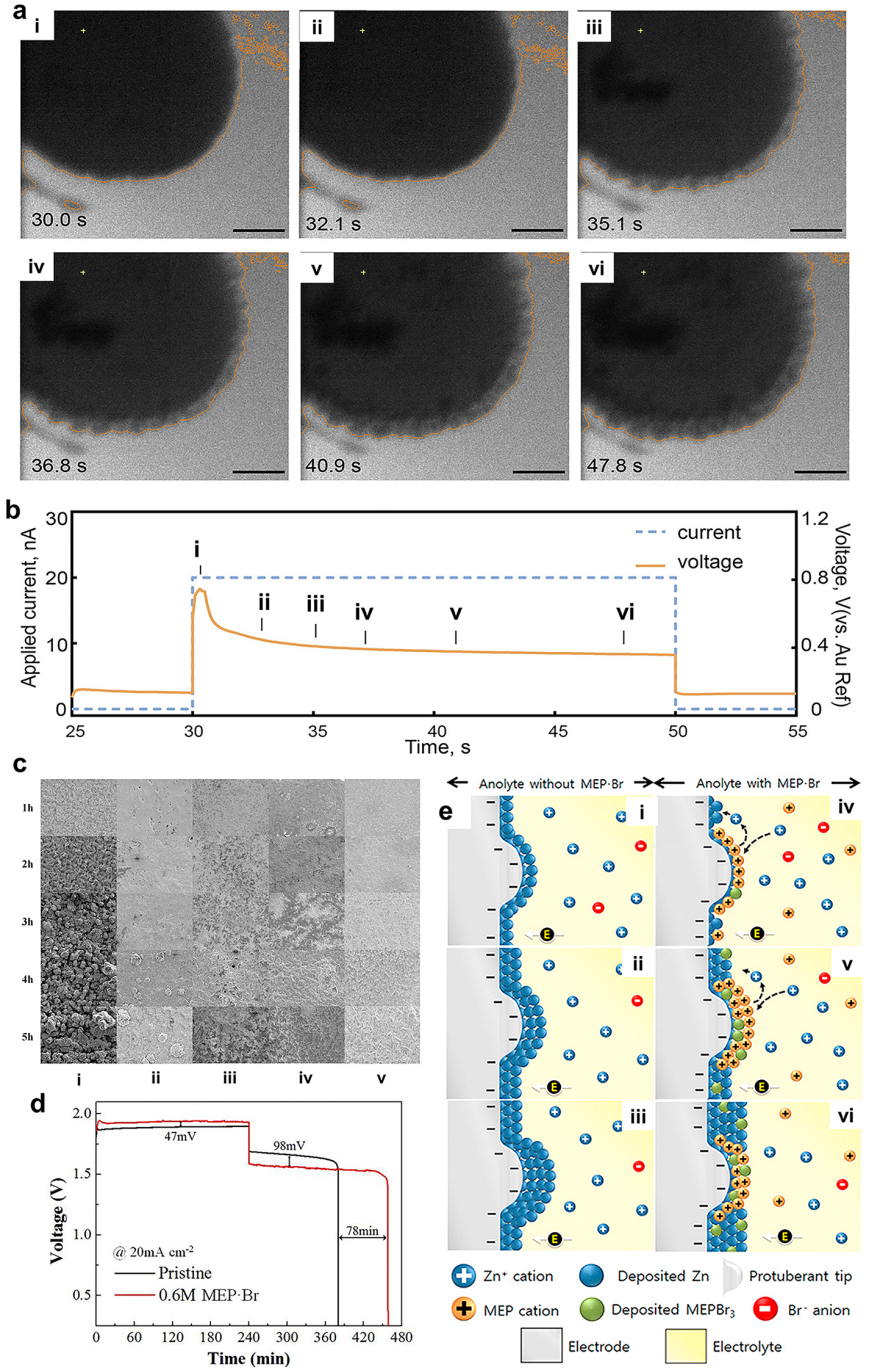
**a** Typical zinc electrodeposition processes with the corresponding voltage response in the in situ EC-LPTEM test: (i–viii) image segmentations of a typical dendrite growth process of zinc electrodeposition and **b** the corresponding voltage response when applying the current during the zinc electrodeposition. The scale bar is 2 μm. Panels **a** and **b** reproduced with permission from Ref. [137]. Copyright 2021, American Chemical Society. **c** × 100 magnification morphologies of deposited zinc due to charge time and MEP∙Br concentrations for a given 2.0 M zinc–bromide electrolyte solution: (i) pristine, (ii) 0.3 M MEP∙Br, (iii) 0.6 M MEP∙Br, (iv) 0.9 M MEP∙Br and (v) 1.2 M MEP∙Br. **d** Charge–discharge curves (capacity vs. potential) of 5th cycle for pristine and 0.6 M MEP∙Br supported electrolyte, and **e** illustration of (i–iii) zinc dendrite growth process (in the case of pristine) and (iv–vi) electrostatic shielding process by MEP cations. Panels **c**–**e** reproduced with permission from Ref. [122]. Copyright 2019, Elsevier B.V

In situ electrochemical liquid phase transmission electron microscopy (EC-LPTEM) can be a useful tool for understanding the characteristics of dendritic growth. Li et al. [137] examined the effect of electrolyte supply flow rate and applied current on the initial stage of dendrites in a zinc half-cell system using in situ EC-LPTEM, as shown in Fig. 10a, b. The authors found that a higher applied current led to longer incubation times for zinc dendrites, suggesting a need to improve the design of electrodes and parameter selection to mitigate the dendrite formation in ZBRBs. While some representative information can be obtained using such instruments, the need for special probing systems with modified battery structures may introduce significant deviations from normal operating conditions for commercial batteries. Alternatively, another study used in situ transmission X-ray microscopy (TXM) as an efficient tool for detecting dendrite formation in minimal architecture zinc–bromine batteries [138]. Numerous details (structure, size, pores, chemical information, etc.) of a 3D morphological dendrite growth were visualised on a carbon cloth electrode immersed in a ZnBr_2_ electrolyte with different organic additives. With these findings, other operando measurements could be developed to detect dendrites and obtain more representative information for optimising the cycling performance in ZBRBs.

In situ reflection absorption Fourier transform infrared (FTIR) spectroscopy is a powerful technique for probing the surface chemistry of carbon surfaces, that provides information on surface oxide species and their potential dependence. This technique can be incorporated with small spot X-ray photoelectron spectroscopy (XPS) as complementary technique to gain detailed information on the chemical composition and electronic structure of the surface. Kautek et al. [139] investigated the electrochemical double layer at the interface with a glassy carbon (GC) electrode and the anodic storage reactions of the polybromide oily phase with two BCAs (i.e. MEP and MEM) in ZBRBs. The authors detected different oxidised surface species including C=O, COO^−^ and C–OH which could be restored at different potentials at the GC electrode. This study also suggested a fast chemical reaction between MEP^+^ cations and Br^−^ anions resulting in MEP-polybromide, while a slow conversion process of the adsorbed MEM-Br ion pairs to the polybromide was determined with a stronger chemical affinity to the GC electrode. A strong interaction was claimed between MEM^+^ morpholinium bridge oxygen and the oxidised species on the surface through hydrogen bridges and/or adsorbed water layers in the absence of ZnBr_2_ in the electrolyte. Based on the study’s outcomes, it was claimed that the efficiency of ZBRBs can be improved when increasing the MEP^+^ concentration in ZnBr_2_ electrolytes. Another study also indicated that the electrochemical performance of zinc plating/stripping during the charge/discharge process was affected by BCAs [122]. In this research, high concentration of MEP∙Br species in the electrolyte improved the zinc plating behaviour and redox reaction reversibility owing to the electrostatic shield effect produced by MEP^+^ cations as shown in Fig. 10c–e. However, real-time observations of ZBRBs that combined with other operando characteristic techniques should be conducted at different operation conditions and electrode materials to deeply understand the interface reactions and the bromine storage complex within the system.

Various measurements and techniques have been also proposed for ex situ and in situ characterisations of RFB electrolytes. For instance, the concentrations of the redox-active species in a ZBFB electrolyte solution can be measured using potentiometric titration as a reliable and accurate ex situ method [140]. However, this method is time-consuming and inappropriate for in situ monitoring when determining the SoC of the RFB electrolyte [140]. Additionally, the estimation of the SoC of the electrolyte should be accurately measured due to its importance to preventing overcharging, discharging and side reactions. Several approaches (e.g. open-circuit voltage method, electrolyte conductivity measurement) have been adopted to analyse the electrolyte's SoC in real time for ZBRBs [121]. Another method that measures electrolyte species concentrations both in situ and ex situ is ultraviolet (UV)/visible (vis) spectroscopy, which is also common for SoC measurements in vanadium-based and organic electrolyte flow batteries [141, 142]. However, it is difficult to obtain accurate SoC estimation in ZBFBs as the active materials exist in a two-phase hybrid system and several equilibrium reactions take place simultaneously, including charge transfer, polybromide formation and complexation [143]. Consequently, more studies are needed to investigate suitable alternatives to improve the SoC analysis in ZBFBs. Lee et al. [121] performed in situ Raman spectroscopy on the negative electrolyte of a single ZBRB cell to estimate the SoC in real time. The authors reported that the total concentration and the SoC of the zinc–bromide electrolyte were strongly correlated with the Raman band intensity observed at about 198 cm^−1^. While this approach can be effective for real-time SoC estimation on the negative electrolyte in ZBRB, it is not quantitatively suitable for the positive electrolyte with the oily polybromide complex phase that disperses non-homogeneously, since it results in significant error, as determined by this study. Therefore, reliable and accurate approaches for determining stability and the SoC of the electrolyte in each half-cell of ZBRBs are highly recommended for better investigations of ZBRBs.

Cycling stability is another major characteristic of ZBRBs that can be measured by capacity fade rate over time (% day) or by cycle number (% cycle). The relative importance of these metrics depends on the most critical degradation mechanism of ZBRBs [39]. For each cycle, CE provides a useful measurement of charge reversibility [144]. A capacity-related process can contribute to CE loss and a non-capacity-related process. Hence, there is no direct correlation between CE and capacity retention (or fade). Reporting and analysing both CE and capacity fade rates are essential, and it is critical to achieve hundreds or thousands of stable cycles in realistic high-energy batteries [144, 145].

In an ideal cell, the CE equals 100%. However, electrolyte–electrode side reactions (chemical or electrochemical) are prevalent in realistic cells. Depending on the reaction pathways, electrons generated by parasitic reactions may or may not be collected by current collectors in the former case [144]. In the latter case, electrons are still lost and accepted by current collectors but are irreversible [144]. Decomposition of electrolytes (e.g. water-splitting reactions including HER and oxygen evolution reaction [OER]) causes electrolyte polymerisation that could lead to low CE, capacity imbalance and irreversible by-products that are not electrochemically active [20, 146]. Current collectors can collect and release electrons in irreversible electrochemical reactions and count them in CE calculations, which complicates the interpretation of the results. Since HER and OER decomposition processes are voltage dependent, choosing a cycling voltage cut-off that minimises HER and OER is essential for revealing RFB intrinsic stability [20]. The capacity fade rate can be calculated using time-dependent and cycle-denominated metrics if the cut-off voltages overlap or exceed HER and/or OER potentials during cycling [20]. To calculate battery efficiencies, amp hours (coulombic) and watt hours (energy) from the battery are measured until reaching the cut-off voltage (e.g. 1.0 V/cell) [71]. After this point, any residual capacity remaining in the battery (unevenly deposited zinc and bromine retained in the cathode activation layer) is considered a residual loss [71]. Transport losses, such as bromine diffusion across the separator and shunt current, can also occur during battery cycling. Energy efficiency is greatly affected by IR losses, as is the case with other battery systems [71]. Other critical performance parameters can be evaluated in ZBRBs, such as voltaic and energy efficiencies.

The volumetric capacity is also a critical parameter for the electrolyte limited by the volumetric capacity of the solid electrode in a solid-hybrid RFB (e.g. ZBFB), where the practically achieved solid electrode capacity is lower than the available liquid electrode capacity. Suppose a solid plating (e.g. zinc plating) reaction cannot progress due to certain limitations (e.g. dendrites, electrode passivation). In that case, it becomes the limiting factor for total cell capacity, even if more capacity is available in the liquid electrolyte. Consequently, the area capacity of the solid reaction is an important factor in evaluating solid-hybrid RFBs (e.g. ZBFBs) and should be controlled to decrease the likelihood of biased comparisons [147, 148].

**Fig. 11:**
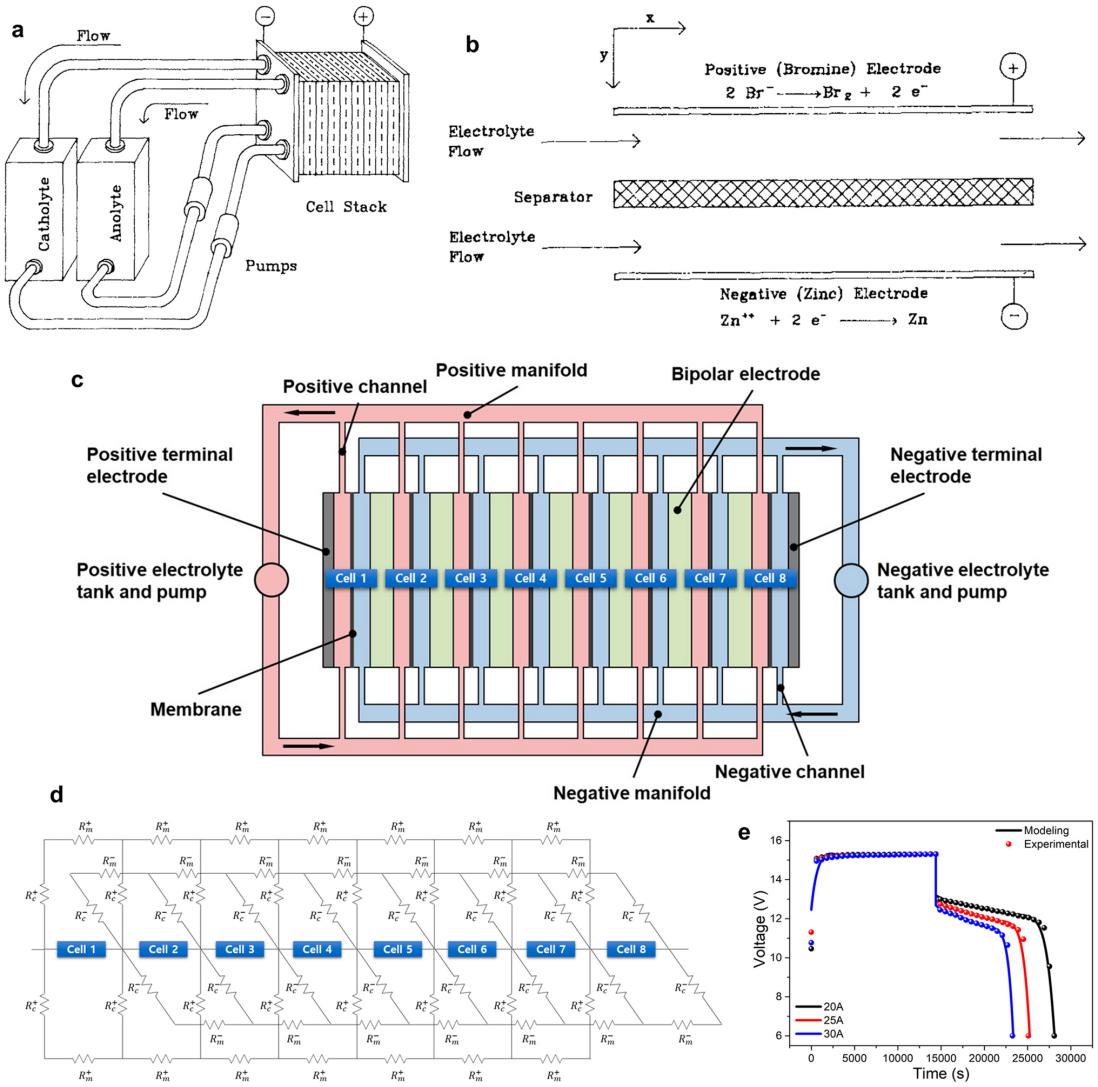
**a** Schematic of a Zn–Br_2_ flow battery and **b** Schematic of the Zn–Br_2_ cell modelled by Lee and Selman (Eqs. 9 and 10), Lee (Eq. 11). Panels **a** and **b** reproduced with permission from Ref. [149]. Copyright 1987, The Electrochemical Society. **c** Schematic of a Zn–Br_2_ flow battery stack composed of 8 cells. **d** An equivalent circuit model corresponding to the schematic of (**c**). **e** Comparison of the modelling charge and discharge behaviours of a Zn–Br_2_ flow battery stack composed of 8 cells with the experimental data. Panels **c**–**e** reproduced with permission from Ref. [151] Copyright 2019, MDPI

Alternatively, the performance of ZBRBs can be assessed by mathematical models that provide great understanding of different design criteria and physical aspects such as the electrochemical processes, cell geometry, operating conditions and species transport affecting the performance of ZBRBs [96, 149]. The choice of a model depends on various factors, such as the thickness of the separator, the initial concentration of the electrolyte and the flow rate of the electrolyte. In more details, the mass transport of species incorporates with migration and diffusion within the electrolyte can be modelled based on various aspects such as flow rate, concentration and ionic conductivity [149]. Moreover, the redox reactions (oxidation and reduction) of zinc and bromine ions can be theoretically formulated to understand the reaction rates occur at the electrode surface and the SEI as well as to predict the system performance along with different experimental work [149]. Current, voltage and energy efficiencies can also be calculated upon different charge and discharge conditions. Different models were developed according to the recirculation system and the parallel plate geometry of a single zinc–bromine flow cell shown in Fig. 11a and b. Based on these cell models, Lee and Selman (Eqs. 9 and 10, respectively) speculated different characteristics considered by some designers of ZBFBs [150]. In these predictions, the current density distribution over the electrode surfaces was modelled besides the overall battery efficiency. By extending Lee and Selman's model, Lee (Eq. 11) has obtained a rough time-dependent current density distribution. To reduce dendrite growth rates in the flow system, he linked his macroscopic model with a microscopic model describing zinc dendrite nucleation and growth and providing important information for improving current density distributions.

9 $$\frac{{\partial c_{i} }}{\partial t} = - \nabla \cdot N_{i } + R_{i }$$

10 $$N_{i } = vc_{i } - D_{i } \nabla c_{i } - \frac{{D_{i } }}{RT}F_{i } \nabla \Phi$$

11 $$i_{j } = i_{{oj.{\text{ref}}\left\{ {\Pi_{i} (\theta_{i,0} )^{pij} \exp \left( {\frac{{ - \partial_{aj} F}}{RT} \eta_{j} } \right) - \Pi_{i} \left( {\theta_{i,0} } \right)^{qij} \exp \left( {\frac{{ - \alpha_{aj} F}}{RT} \eta_{j} } \right)} \right\}}}$$

where $\frac{{\partial c_{i} }}{\partial t}$ is the accumulation term and set to be equal to zero, $N_{i }$ is the species flux, $R_{i }$ production rate of species *i* due to reaction, *v* is the electrode potential (V), $c_{i }$ is the concentration of species *i* (mol cm^−3^), $D_{i }$ is the diffusion coefficient of species *i* (cm^2^ s^−1^), *i* is the current density (A m^−2^), $\alpha_{aj}$ is the anodic transfer coefficient for reaction *j*, $i_{j }$ is the faradaic current density due to electrochemical reaction *j*, $\theta_{i,0}$ is the dimensionless local surface concentration of species *I*, $\eta_{j}$ is the overpotential for reaction *j* at electrode surface, $i_{{oj.{\text{ref}}}}$ is the exchange current density of electrochemical reaction *j* and *F* Faraday's constant (96,487 C mol^−1^).

In addition, Koo et al. [151] reported a simple modelling approach to demonstrate the electrochemical behaviour of ZBFB of an 8-cell Zn–Br_2_ stack with an equivalent circuit model employing the inlet and outlet resistances of the positive and negative electrolytes as shown in Fig. 11c, d. Based on simplified polarisation characteristics of the electrodes, Ohm's law and charge conservation were used to model charge and discharge behaviour of a single cell in this study. The modelling results ware validated when compared with the experimental measurements as shown in Fig. 11e. Shah et al. [152] developed a mathematical model to study of hydrogen evolution during charge in the negative half-cell of an all-vanadium redox flow battery, which tied together the dynamic conservation equations for charge, mass, and momentum, as well as detailed descriptions of the electrochemical reactions. Using a modified multiphase-mixture approach, the model included bubble formation at the negative electrode, considering the attendant reduction in liquid volume and momentum transfer between gas and liquid. Several vanadium concentrations and electrolyte flow rates were simulated numerically with good agreement with experimental data. In this study, simulations with negligible hydrogen evolution demonstrated that gas evolution impacted the battery efficiency which was mainly due to partial current consumption for driving the H_2_ reaction, reducing the negative electrode's current density associated with the redox reactions. It was also predicted that the gas volume fraction and the bubble velocity could significantly vary according to the operating conditions.

Although modelling and simulation tools are supportive and cost-effective methods for reducing the number of test cases, existing models of the ZBRBs do not account for all of the battery's features. Therefore, developing more theoretical research tools with different cell features in the model besides considering other model tools like computational models to identify complex systems and simulate real world experiment are important to obtain fundamental knowledge regarding various phenomena in the ZBRB systems. The model should also have simplifying assumptions for a realistic and useful prediction for cell designers in the industry sectors.

While the electrodes do not participate in the electrochemical reactions, they are susceptible to oxidation degradation during charge/discharge cycles. Their degradation can reduce surface activity and potentially affect the mechanical integrity of the electrode core. Understanding the material characteristics and operational conditions that lead to degradation can inform the selection of superior component formulations and operating conditions, thereby extending the operational life of the ZBRBs. In addition, the selection of appropriate electrode material depends on several factors. First, the electrochemical properties of the electrode materials (e.g. titanium or platinum) that can withstand high temperatures and corrosive environments. Moreover, a high conductive electrode material (e.g. gold and silver) is important as it affects how well electrical current flows through it. These materials are often considered in applications where low resistance is required. The stability and cost of the electrode are further requirements that should be considered when selecting appropriate materials. However, some materials may outperform others while still meeting the preferred requirements for battery performance. Therefore, more studies and assessment of different electrodes are highly required when selecting appropriate materials to achieve improved ZBRB systems.

All assessment methods, tools and performance metrics summarised in Table 2 can be used to evaluate the performance and cost-effectiveness of zinc–bromine batteries and compare them to other energy storage technologies. By optimising these metrics through advanced configurations and research, zinc–bromine batteries can become a more competitive and sustainable solution for energy storage.

**Table 2:** Summary of performance metrics, cell attributes and assessment methods of ZBRBs

	Description	Significance and definition
*Performance metrics and cell attributes*		
Cycling stability	The ability of a battery to maintain its capacity and performance over multiple charge and discharge cycles	Both cycling life and stability indicate the battery degradation mechanism
Cycle life	The number of charge and discharge cycles that the battery can withstand before its capacity degrades below a certain threshold	
Coulombic efficiency (CE)	A measure of how much of the energy charged into a battery is available for use during discharge	Provides a useful measurement of charge reversibility CE = *Q*_discharge_/*Q*_charge_ × 100 [144]
Voltaic efficiency (VE)^a^	A measure of how much energy is available from a battery compared to what was charged into the battery	VE = $\overline{E}_{{{\text{disch}}}} /\overline{E}_{{{\text{ch}}}}$ × 100 [153]
Energy efficiency (EE)	The amount of energy the battery can deliver relative to the amount of energy it consumes during charging and discharging	EE = *E*_*v*,cell discharge_*/E*_*v*,cell charge_ × 100 [153]
Power density^b^	The rate at which the battery can deliver energy	*P* = (*I* × *V*_cell_)/*A* [20]
Energy density	The amount of energy that the battery can store relative to its weight	*E*_*v*,cell_ = $\frac{{\int {{\text{d}}Q_{{{\text{discharge}}}} { } \times {\text{ d}}V_{{{\text{cell}}}} } }}{{V_{ + } + V_{ - } }}$ [154]
Theoretical electrolyte capacity^c^	An electrolyte's theoretical capacity stored in a given volume	*Q*_*t*_ = *n* × *c* × *v* × *F*/3600 = *ncv* × 26.8 (mAh) [20]
SoC	The charged capacity stored over the calculated theoretical capacity	SoC = *Q*_charge_/*Q*_*t*_ [155]
	Output and applicability	Challenges
*Assessment methods*		
Electrochemical liquid phase transmission electron microscopy (EC-LPTEM)	Understanding the mechanism of the dendritic growth In situ	Commercial batteries may experience significant deviations from normal operating conditions when using special probing systems with modified battery structures
Transmission X-ray microscopy (TXM)	Detecting dendrite formation in minimal architecture zinc–bromine batteries In situ	Requires special cell structure to be used for in situ measurements
Potentiometric titration	Detecting the concentrations of the redox-active species in ZBRB electrolyte solutions Ex situ	Time-consuming
UV/vis spectroscopy	Measuring the electrolyte species concentrations Determining the SoC of the RFB electrolyte In situ and ex situ	Difficult to obtain accurate SoC estimation value in ZBRBs as the active materials exist in a two-phase hybrid system
Raman spectroscopic analysis	Detecting the total concentration and the SoC of the zinc–bromide electrolyte for the real-time SoC estimation of the negative electrolyte in ZBRBs In situ	Not quantitatively suitable for the positive electrolyte with the oily polybromide complex phase that is dispersed non-homogeneously
Fourier transform infrared (FTIR) spectroscopy	Determining the electrochemical double layer at the SEI on carbon-based materials (e.g. glassy carbon) Determining the storage complex reactions and rates of some organic BCAs (e.g. MEP and MEM) In situ	Requires more studies on different carbonous electrodes (e.g. graphite and carbon felt) with different properties (e.g. surface area)
X-ray photoelectron spectroscopy (XPS)	Providing detailed information on the chemical composition and electronic structure of the surface Ex situ	Requires special cell structure to be used for in situ measurements

^a^ $\overline{E}_{{{\text{disch}}}}$ average voltage over discharge, $\overline{E}_{{{\text{ch}}}}$ average voltage over charge

^b^*I* is current (mA) and *V*_cell_ is cell voltage at a given current (V)

^c^*n* is the number of electrons per mole, *c* is concentration (mol L^−1^), *v* is volume (mL) and *F* is the Faraday constant

## Conclusion and Perspectives

**Fig. 12:**
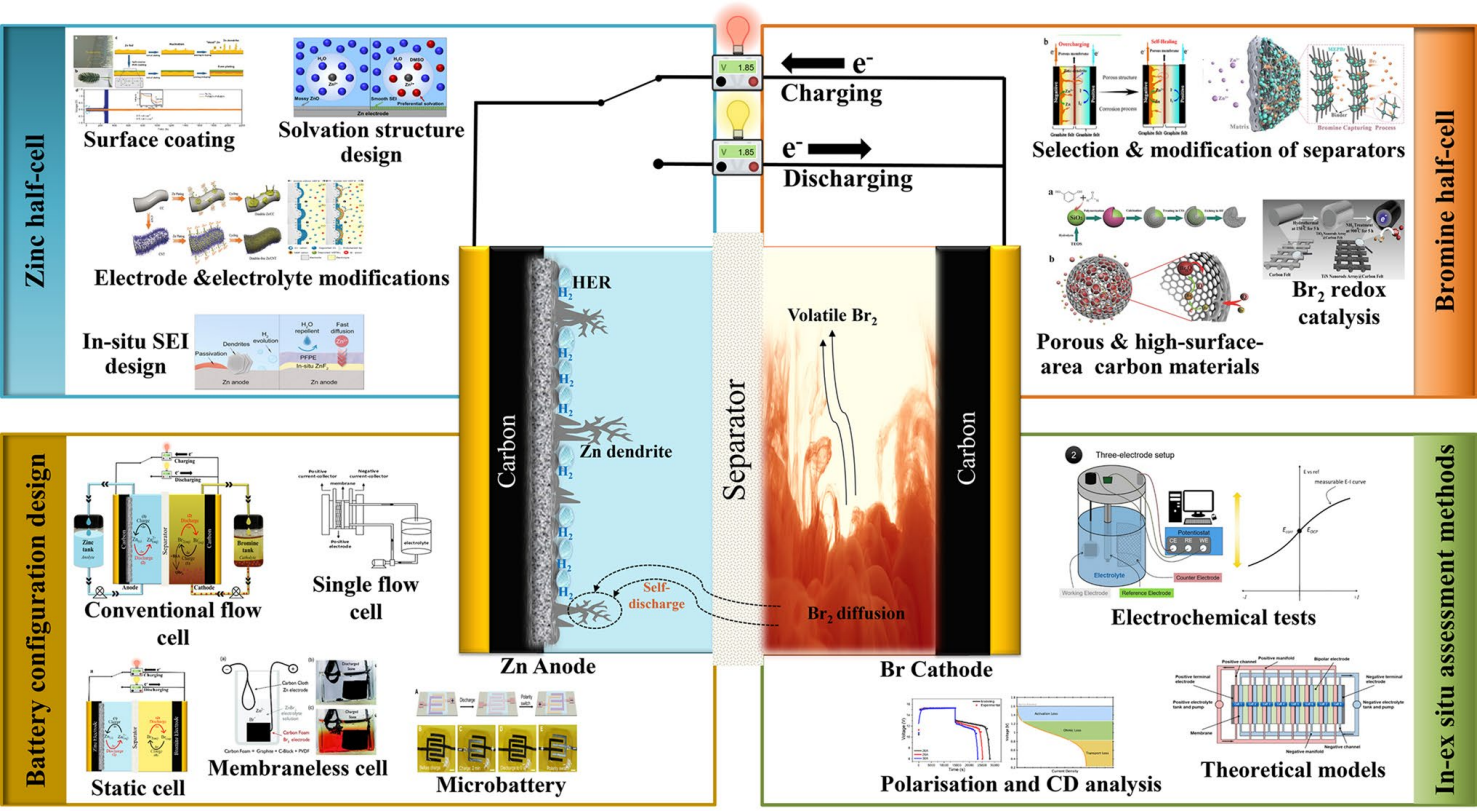
Schematic illustration of ZBRBs from device configuration, electrochemistry, material to performance evaluation

Zinc–bromine rechargeable batteries are a promising candidate for stationary energy storage applications due to their non-flammable electrolyte, high cycle life, high energy density and low material cost. Different structures of ZBRBs have been proposed and developed over time, from static (non-flow) to flowing electrolytes. Nevertheless, ZBRBs still need to overcome certain challenges to accelerate their commercialisation. As illustrated in Fig. 12, in this review, we initially discussed various cell configurations of ZBRBs, as well as their unique features and components. The electrochemistry of ZBRBs was also highlighted with a focus on chemical processes occurring at the zinc and bromine electrodes, such as zinc deposition and stripping reactions, the bromine reaction mechanism and the formation of polybromide quaternary ammonium salt. The challenges pertaining to both electrodes were also reviewed, including dendrite formation and HER at the negative electrode and the sluggish kinetics of the bromine redox couples at the positive electrode. Achieving uniform and dense zinc deposition while suppressing hydrogen evolution translates into higher capacity, higher efficiency and a longer cycle life. The bromine sequestration agent and membrane play an important role in preventing the crossover of bromine-bearing species. To facilitate future ZBRB benchmarking and development, different assessment methods and performance metrics of this technology were also discussed in this work. While various promising strategies have been developed to increase the knowledge about and improve the electrochemical and physical processes of zinc–bromine battery systems, the current strategies have proven insufficient to boost the state of this technology, and more research must be conducted to further improve this technology and overcome the challenges such that it is more reliable for the future market. It is expected that this review will contribute to a better understanding of ZBRB systems, assisting and guiding future research and providing a general framework for future efforts. An analysis of the latest developments shows that, despite the progress made for the system improvement and stability, much remains to be done. Therefore, we propose the following suggestions:

- Significant improvements to zinc–bromine system performance can be made by implementing advanced configurations to make them more competitive with other energy storage technologies. Continued research and development efforts in this area are critical to advance the field of energy storage and meet the increasing demand for reliable and sustainable energy solutions.
- Using alternative functionalised surface materials and understanding the interfacial mechanism at the zinc electrode will improve smooth zinc plating morphology, suppressing hydrogen evolution during the charging process and ensuring full zinc stripping out during discharge.
- The sluggish kinetics of Br_2_/Br^−^ redox can be enhanced by improving strategies and/or adapting existing methods, such as using highly conductive carbon materials with a high surface area to catalyse the bromine couple. More studies on understanding the bromine reaction mechanism and fabrication of advanced structures can also improve the bromine electrode and thus improve the zinc–bromine system performance.
- With further testing and understanding from fundamental studies, different methods and electrochemical techniques, promising investigative pathways could become clearer, and developing potential solutions would take less time and effort. Electrochemical techniques can also assist in identifying alternative physical configurations and determining the nature and degree of functionality of future electrode materials. Therefore, the cost would be reduced while the operational efficiency and practical specific energy would be improved, thus reducing adverse side reactions.

In conclusion, a significant understanding of the physical and electrochemical processes governing ZBRB operation during charge and discharge processes is required to develop the next generation of ZBRBs. Using this understanding, we could formulate and select optimal stack configurations, specific electrode functionalisation, useful functional additives to employ in the electrolyte. These developments would direct further research avenues, possibly leading to modification of the ZBRB design itself.

## References

[1] Gür TM (2018). Review of electrical energy storage technologies, materials and systems: challenges and prospects for large-scale grid storage. Energy Environ. Sci..

[2] Leung P, Li XH, de Leon CP, Berlouis L, Low CTJ (2012). Progress in redox flow batteries, remaining challenges and their applications in energy storage. RSC Adv..

[3] Chen C, Lee CS, Tang Y (2023). Fundamental understanding and optimization strategies for dual-ion batteries: a review. Nano-Micro Lett..

[4] Yang J, Yin B, Sun Y, Pan H, Sun W (2022). Zinc anode for mild aqueous zinc-ion batteries: challenges, strategies, and perspectives. Nano-Micro Lett..

[5] Li X, Chen F, Zhao B, Zhang S, Zheng X (2023). Ultrafast synthesis of metal-layered hydroxides in a dozen seconds for high-performance aqueous Zn (micro-) battery. Nano-Micro Lett..

[6] Castillo A, Gayme DF (2014). Grid-scale energy storage applications in renewable energy integration: a survey. Energy Convers. Manag..

[7] Ahmed S, Mahmood A, Hasan A, Sidhu GAS, Butt MFU (2016). A comparative review of china, india and pakistan renewable energy sectors and sharing opportunities. Renew. Sustain. Energy Rev..

[8] Wu C, Tong X, Ai Y, Liu DS, Yu P (2018). A review: enhanced anodes of Li/Na-ion batteries based on yolk–shell structured nanomaterials. Nano-Micro Lett..

[9] Wang W, Luo QT, Li B, Wei XL, Li LY (2013). Recent progress in redox flow battery research and development. Adv. Funct. Mater..

[10] Ye RJ, Henkensmeier D, Yoon SJ, Huang ZF, Kim DK (2018). Redox flow batteries for energy storage: a technology review. J. Electrochem. Energy Convers. Storage.

[11] Gao L, Li Z, Zou Y, Yin S, Peng P (2020). A high-performance aqueous zinc-bromine static battery. iScience.

[12] Biswas S, Senju A, Mohr R, Hodson T, Karthikeyan N (2017). Minimal architecture zinc-bromine battery for low cost electrochemical energy storage. Energy Environ. Sci..

[13] Barnartt S, Forejt DA (1964). Bromine-zinc secondary cells. J. Electrochem. Soc..

[14] Butler PC, Eidler PA, Grimes PG, Klassen SE, Miles RC, By Linden D, Reddy T (2001). Handbook of Batteries.

[15] Dai C, Hu L, Jin X, Wang Y, Wang R (2022). Fast constructing polarity-switchable zinc-bromine microbatteries with high areal energy density. Sci. Adv..

[16] Plackett B (2021). A conversation with thomas maschmeyer. ACS Cent. Sci..

[17] Lee JH, Byun Y, Jeong GH, Choi C, Kwen J (2019). High-energy efficiency membraneless flowless Zn–Br battery: utilizing the electrochemical-chemical growth of polybromides. Adv. Mater..

[18] Chen XW, Hopkins BJ, Helal A, Fan FY, Smith KC (2016). A low-dissipation, pumpless, gravity-induced flow battery. Energy Environ. Sci..

[19] Braff WA, Bazant MZ, Buie CR (2013). Membrane-less hydrogen bromine flow battery. Nat. Commun..

[20] Yao YX, Lei JF, Shi Y, Ai F, Lu YC (2021). Assessment methods and performance metrics for redox flow batteries. Nat. Energy.

[21] K. Likit-anurak, K. Uthaichana, K. Punyawudho, Y. Khunatorn, The performance and efficiency of organic electrolyte redox flow battery prototype, in *2017 2nd International Conference on Advances on Clean Energy Research (Icacer 2017)*, vol. 118 (2017), pp. 54–62. 10.1016/j.egypro.2017.07.012

[22] Mourshed M, Niya SMR, Ojha R, Rosengarten G, Andrews J (2021). Carbon-based slurry electrodes for energy storage and power supply systems. Energy Storage Mater..

[23] Lourenssen K, Williams J, Ahmadpour F, Clemmer R, Tasnim S (2019). Vanadium redox flow batteries: a comprehensive review. J. Energy Storage.

[24] Cunha A, Martins J, Rodrigues N, Brito FP (2015). Vanadium redox flow batteries: a technology review. Int. J. Energy Res..

[25] Houser J, Pezeshki A, Clement JT, Aaron D, Mench MM (2017). Architecture for improved mass transport and system performance in redox flow batteries. J. Power Sources.

[26] Suresh S, Kesavan T, Munaiah Y, Arulraj I, Dheenadayalan S (2014). Zinc-bromine hybrid flow battery: effect of zinc utilization and performance characteristics. RSC Adv..

[27] De Leon CP, Frías-Ferrer A, González-García J, Szánto D, Walsh FC (2006). Redox flow cells for energy conversion. J. Power Sources.

[28] Skyllas-Kazacos M, Chakrabarti MH, Hajimolana SA, Mjalli FS, Saleem M (2011). Progress in flow battery research and development. J. Electrochem. Soc..

[29] Parasuraman A, Lim TM, Menictas C, Skyllas-Kazacos M (2013). Review of material research and development for vanadium redox flow battery applications. Electrochim. Acta.

[30] L.H. Thaller, Electrically rechargeable redox flow cells, in *9th Intersociety Energy Conversion Engineering Conference* (1974), pp. 924–928

[31] Wang ZY, Tam LYS, Lu YC (2019). Flexible solid flow electrodes for high-energy scalable energy storage. Joule.

[32] Park M, Ryu J, Wang W, Cho J (2016). Material design and engineering of next-generation flow-battery technologies. Nat. Rev. Mater..

[33] Zeng YK, Zhou XL, An L, Wei L, Zhao TS (2016). A high-performance flow-field structured iron-chromium redox flow battery. J. Power Sources.

[34] Gong K, Ma XY, Conforti KM, Kuttler KJ, Grunewald JB (2015). A zinc-iron redox-flow battery under $100 per kWh of system capital cost. Energy Environ. Sci..

[35] Li ZJ, Weng GM, Zou QL, Cong GT, Lu YC (2016). A high-energy and low-cost polysulfide/iodide redox flow battery. Nano Energy.

[36] Wang C, Lai Q, Xu P, Zheng D, Li X (2017). Cage-like porous carbon with superhigh activity and Br(2)-complex-entrapping capability for bromine-based flow batteries. Adv. Mater..

[37] Wei L, Zhao TS, Zeng L, Zhou XL, Zeng YK (2016). Copper nanoparticle-deposited graphite felt electrodes for all vanadium redox flow batteries. Appl. Energy.

[38] Minke C, Turek T (2018). Materials, system designs and modelling approaches in techno-economic assessment of all-vanadium redox flow batteries—a review. J. Power Sources.

[39] Kwabi DG, Ji Y, Aziz MJ (2020). Electrolyte lifetime in aqueous organic redox flow batteries: a critical review. Chem. Rev..

[40] Ke X, Prahl JM, Alexander JID, Wainright JS, Zawodzinski TA (2018). Rechargeable redox flow batteries: flow fields, stacks and design considerations. Chem. Soc. Rev..

[41] Kim KJ, Park MS, Kim YJ, Kim JH, Dou SX (2015). A technology review of electrodes and reaction mechanisms in vanadium redox flow batteries. J. Mater. Chem. A.

[42] Joerissen L, Garche J, Fabjan C, Tomazic G (2004). Possible use of vanadium redox-flow batteries for energy storage in small grids and stand-alone photovoltaic systems. J. Power Sources.

[43] Luo JA, Hu B, Hu MW, Zhao Y, Liu TL (2019). Status and prospects of organic redox flow batteries toward sustainable energy storage. ACS Energy Lett..

[44] Winsberg J, Hagemann T, Janoschka T, Hager MD, Schubert US (2017). Redox-flow batteries: from metals to organic redox-active materials. Angew. Chem. Int. Ed..

[45] Singh V, Kim S, Kang J, Byon HR (2019). Aqueous organic redox flow batteries. Nano Res..

[46] Janoschka T, Martin N, Martin U, Friebe C, Morgenstern S (2015). An aqueous, polymer-based redox-flow battery using non-corrosive, safe, and low-cost materials. Nature.

[47] Huskinson B, Marshak MP, Suh C, Er S, Gerhardt MR (2014). A metal-free organic–inorganic aqueous flow battery. Nature.

[48] Chen HN, Cong GT, Lu YC (2018). Recent progress in organic redox flow batteries: active materials, electrolytes and membranes. J. Energy Chem..

[49] Zeng YK, Zhao TS, An L, Zhou XL, Wei L (2015). A comparative study of all-vanadium and iron-chromium redox flow batteries for large-scale energy storage. J. Power Sources.

[50] Zeng YK, Zhao TS, Zhou XL, Zeng L, Wei L (2016). The effects of design parameters on the charge–discharge performance of iron-chromium redox flow batteries. Appl. Energy.

[51] Sun CY, Zhang H (2019). Investigation of nafion series membranes on the performance of iron-chromium redox flow battery. Int. J. Energy Res..

[52] Chang SL, Ye JY, Zhou W, Wu C, Ding M (2019). A low-cost speek-k type membrane for neutral aqueous zinc-iron redox flow battery. Surf. Coat. Technol..

[53] Yuan Z, Duan Y, Liu T, Zhang H, Li X (2018). Toward a low-cost alkaline zinc-iron flow battery with a polybenzimidazole custom membrane for stationary energy storage. iScience.

[54] Amini K, Pritzker MD (2020). In situ polarization study of zinc-cerium redox flow batteries. J. Power Sources.

[55] D.P. Trudgeon, K.P. Qiu, X.H. Li, T. Mallick, O.O. Taiwo et al., Screening of effective electrolyte additives for zinc-based redox flow battery systems. J. Power Sources **412**(44–54 (2019). 10.1016/j.jpowsour.2018.11.030

[56] Arenas LF, Loh A, Trudgeon DP, Li XH, de Leon CP (2018). The characteristics and performance of hybrid redox flow batteries with zinc negative electrodes for energy storage. Renew. Sustain. Energy Rev..

[57] Soloveichik GL (2015). Flow batteries: current status and trends. Chem. Rev..

[58] Weng GM, Li ZJ, Cong GT, Zhou YC, Lu YC (2017). Unlocking the capacity of iodide for high-energy-density zinc/polyiodide and lithium/polyiodide redox flow batteries. Energy Environ. Sci..

[59] Sánchez-Díez E, Ventosa E, Guarnieri M, Trovò A, Flox C (2021). Redox flow batteries: status and perspective towards sustainable stationary energy storage. J. Power Sources.

[60] Li LY, Kim S, Wang W, Vijayakumar M, Nie ZM (2011). A stable vanadium redox-flow battery with high energy density for large-scale energy storage. Adv. Energy Mater..

[61] Yuan Z, Yin Y, Xie C, Zhang H, Yao Y (2019). Advanced materials for zinc-based flow battery: development and challenge. Adv. Mater..

[62] Sepulveda NA, Jenkins JD, Edington A, Mallapragada DS, Lester RK (2021). The design space for long-duration energy storage in decarbonized power systems. Nat. Energy.

[63] Kim H-T, Lee J-H, Kim DS, Yang JH, Passerini S, Bresser D, Moretti A, Varzi A (2020). Batteries: Present and Future Energy Storage Challenges.

[64] Rajarathnam GP, Ellis TK, Adams AP, Soltani B, Zhou RW (2021). Chemical speciation of zinc-halide complexes in zinc/bromine flow battery electrolytes. J. Electrochem. Soc..

[65] Periyapperuma K, Zhang YF, MacFarlane DR, Forsyth M, Pozo-Gonzalo C (2017). Towards higher energy density redox-flow batteries: imidazolium ionic liquid for Zn electrochemistry in flow environment. ChemElectroChem.

[66] Xu P, Li T, Zheng Q, Zhang H, Yin Y (2022). A low-cost bromine-fixed additive enables a high capacity retention zinc–bromine batteries. J. Energy Chem..

[67] Xu ZC, Fan Q, Li Y, Wang J, Lund PD (2020). Review of zinc dendrite formation in zinc bromine redox flow battery. Renew. Sustain. Energy Rev..

[68] Rajarathnam GP, Vassallo AM (2016). The Zinc/Bromine Flow Battery: Materials Challenges and Practical Solutions for Technology Advancement.

[69] *Accelerating a Carbon-Free Future* (Redflow Limited, 2023). https://redflow.com/. Accessed 2 March 2023

[70] *The Future of Storage is Long* (Primuspower, 2023). https://primuspower.com/en/. Accessed 3 March 2023

[71] P. Lex, J. Matthews, Recent developments in zinc/bromine battery technology at johnson controls, in *IEEE 35th International Power Sources Symposium* (1992), pp. 88–92

[72] Lai QZ, Zhang HM, Li XF, Zhang LQ, Cheng YH (2013). A novel single flow zinc–bromine battery with improved energy density. J. Power Sources.

[73] Jang WI, Lee JW, Baek YM, Park OO (2016). Development of a pp/carbon/cnt composite electrode for the zinc/bromine redox flow battery. Macromol. Res..

[74] Abd El Rehim SS, Abd El Wahaab SM, Fouad EE, Hassan HH (1994). Effect of some variables on the electroplating of zinc from acidic acetate baths. J. Appl. Electrochem..

[75] Ohtaki H, Radnai T (1993). Structure and dynamics of hydrated ions. Chem. Rev..

[76] Guo L, Guo H, Huang H, Tao S, Cheng Y (2020). Inhibition of zinc dendrites in zinc-based flow batteries. Front. Chem..

[77] Wang KL, Pei PC, Ma Z, Chen HC, Xu HC (2015). Dendrite growth in the recharging process of zinc–air batteries. J. Mater. Chem. A.

[78] Jiang HR, Wu MC, Ren YX, Shyy W, Zhao TS (2018). Towards a uniform distribution of zinc in the negative electrode for zinc bromine flow batteries. Appl. Energy.

[79] Zeng Y, Zhang X, Qin R, Liu X, Fang P (2019). Dendrite-free zinc deposition induced by multifunctional CNT frameworks for stable flexible Zn-ion batteries. Adv. Mater..

[80] Sun KE, Hoang TK, Doan TN, Yu Y, Zhu X (2017). Suppression of dendrite formation and corrosion on zinc anode of secondary aqueous batteries. ACS Appl. Mater. Interfaces.

[81] Lu W, Xie C, Zhang H, Li X (2018). Inhibition of zinc dendrite growth in zinc-based batteries. Chemsuschem.

[82] Ortiz-Aparicio JL, Meas Y, Trejo G, Ortega R, Chapman TW (2013). Effects of organic additives on zinc electrodeposition from alkaline electrolytes. J. Appl. Electrochem..

[83] Hao JN, Li XL, Zhang SL, Yang FH, Zeng XH (2020). Designing dendrite-free zinc anodes for advanced aqueous zinc batteries. Adv. Funct. Mater..

[84] Chladil L, Cech O, Smejkal J, Vanysek P (2019). Study of zinc deposited in the presence of organic additives for zinc-based secondary batteries. J. Energy Storage.

[85] Worku AK (2022). Engineering techniques to dendrite free zinc-based rechargeable batteries. Front. Chem..

[86] Chen R, Zhang W, Huang Q, Guan C, Zong W (2023). Trace amounts of triple-functional additives enable reversible aqueous zinc-ion batteries from a comprehensive perspective. Nano-Micro Lett..

[87] Song Y, Ruan P, Mao C, Chang Y, Wang L (2022). Metal-organic frameworks functionalized separators for robust aqueous zinc-ion batteries. Nano-Micro Lett..

[88] Meng H, Ran Q, Dai TY, Shi H, Zeng SP (2022). Surface-alloyed nanoporous zinc as reversible and stable anodes for high-performance aqueous zinc-ion battery. Nano-Micro Lett..

[89] Xie SY, Li Y, Li X, Zhou YJ, Dang ZQ (2022). Stable zinc anodes enabled by zincophilic Cu nanowire networks. Nano-Micro Lett..

[90] Wang YY, Chen YJ, Liu W, Ni XY, Qing P (2021). Uniform and dendrite-free zinc deposition enabled by in situ formed AgZn_3_ for the zinc metal anode. J. Mater. Chem. A.

[91] Li B, Zhang XT, Wang TL, He ZX, Lu BA (2022). Interfacial engineering strategy for high-performance Zn metal anodes. Nano-Micro Lett..

[92] Wu K, Yi J, Liu X, Sun Y, Cui J (2021). Regulating Zn deposition via an artificial solid–electrolyte interface with aligned dipoles for long life Zn anode. Nano-Micro Lett..

[93] Zhang Q, Luan J, Tang Y, Ji X, Wang H (2020). Interfacial design of dendrite-free zinc anodes for aqueous zinc-ion batteries. Angew. Chem. Int. Ed..

[94] Wang X, Li X, Fan H, Ma L (2022). Solid electrolyte interface in Zn-based battery systems. Nano-Micro Lett..

[95] Zhao KN, Wang CX, Yu YH, Yan MY, Wei QL (2018). Ultrathin surface coating enables stabilized zinc metal anode. Adv. Mater. Inter..

[96] Ganne F, Cachet C, Maurin G, Wiart R, Chauveau E (2000). Impedance spectroscopy and modelling of zinc deposition in chloride electrolyte containing a commercial additive. J. Appl. Electrochem..

[97] Xu K (2004). Nonaqueous liquid electrolytes for lithium-based rechargeable batteries. Chem. Rev..

[98] Jiang HR, Shyy W, Wu MC, Wei L, Zhao TS (2017). Highly active, bi-functional and metal-free B4C-nanoparticle-modified graphite felt electrodes for vanadium redox flow batteries. J. Power Sources.

[99] Tian Y, Xu L, Li M, Yuan D, Liu X (2021). Correction to: interface engineering of CoS/COO@n-doped graphene nanocomposite for high-performance rechargeable Zn–air batteries. Nano-Micro Lett..

[100] Jiang J, Nie G, Nie P, Li Z, Pan Z (2020). Nanohollow carbon for rechargeable batteries: ongoing progresses and challenges. Nano-Micro Lett..

[101] Xu X, Xu Y, Zhang J, Zhong Y, Li Z (2023). Quasi-solid electrolyte interphase boosting charge and mass transfer for dendrite-free zinc battery. Nano-Micro Lett..

[102] Wang N, Wan H, Duan J, Wang X, Tao L (2021). A review of zinc-based battery from alkaline to acid. Mater. Today Adv..

[103] R. Putt, *Assessment of Technical and Economic Feasibility of Zinc/Bromine Batteries for Utility Load Leveling*. Final Report Gould (Gould, Inc., Rolling Meadows, 1979). 10.2172/6106713

[104] Wang M, Meng Y, Li K, Ahmad T, Chen N (2022). Toward dendrite-free and anti-corrosion Zn anodes by regulating a bismuth-based energizer. eScience.

[105] Jia H, Qiu MH, Tang CX, Liu HQ, Fu SH (2022). Nano-scale BN interface for ultra-stable and wide temperature range tolerable Zn anode. EcoMat.

[106] Shang W, Li Q, Jiang F, Huang B, Song J (2022). Boosting Zn||I(2) battery's performance by coating a zeolite-based cation-exchange protecting layer. Nano-Micro Lett..

[107] Wu ZX, Yuan XH, Jiang MJH, Wang LL, Huang QH (2020). Zinc–carbon paper composites as anodes for Zn-ion batteries: key impacts on their electrochemical behaviors. Energy Fuels.

[108] Li M, He Q, Li ZL, Li Q, Zhang YX (2019). A novel dendrite-free Mn^2+^/Zn^2+^ hybrid battery with 2.3 V voltage window and 11000-cycle lifespan. Adv. Energy Mater..

[109] Cao L, Li D, Hu E, Xu J, Deng T (2020). Solvation structure design for aqueous Zn metal batteries. J. Am. Chem. Soc..

[110] Zhang C, Shin W, Zhu LD, Chen C, Neuefeind JC (2021). The electrolyte comprising more robust water and superhalides transforms Zn-metal anode reversibly and dendrite-free. Carbon Energy.

[111] Llopis J, Vàzquez M (1962). Study of the impedance of a platinum electrode in the system Br_2_/Br^−^ (HClO4, aq.). I. Influence of the surface state. Electrochim. Acta.

[112] Magno F, Mazzocchin G-A, Bontempelli G (1973). Electrochemical behaviour of the bromide ion at a platinum electrode in acetonitrile solvent. J. Electroanal. Chem. Interfacial Electrochem..

[113] Cathro KJ, Cedzynska K, Constable DC, Hoobin PM (1986). Selection of quaternary ammonium bromides for use in zinc bromine cells. J. Power Sources.

[114] White RE, Lorimer SE (1983). A model of the bromine bromide electrode-reaction at a rotating-disk electrode. J. Electrochem. Soc..

[115] Popat Y, Trudgeon D, Zhang CP, Walsh FC, Connor P (2022). Carbon materials as positive electrodes in bromine-based flow batteries. ChemPlusChem.

[116] Janssen LJJ, Hoogland JG (1970). Mechanism of bromine evolution at a graphite electrode. Electrochim. Acta.

[117] Mastragostino M, Gramellini C (1985). Kinetic study of the electrochemical processes of the bromine/bromine aqueous system on vitreous carbon electrodes. Electrochim. Acta.

[118] Wu WL, Xu SC, Lin ZR, Lin L, He RH (2022). A polybromide confiner with selective bromide conduction for high performance aqueous zinc–bromine batteries. Energy Storage Mater..

[119] Yu F, Pang L, Wang XX, Waclawik ER, Wang FX (2019). Aqueous alkaline–acid hybrid electrolyte for zinc–bromine battery with 3 V voltage window. Energy Storage Mater..

[120] Lancry E, Magnes B-Z, Ben-David I, Freiberg M (2013). New bromine complexing agents for bromide based batteries. ECS Trans..

[121] Lee HJ, Kim DW, Yang JH (2017). Estimation of state-of-charge for zinc–bromine flow batteries by in situ raman spectroscopy. J. Electrochem. Soc..

[122] Kim M, Yun D, Jeon J (2019). Effect of a bromine complex agent on electrochemical performances of zinc electrodeposition and electrodissolution in zinc–bromide flow battery. J. Power Sources.

[123] Archana KS, Naresh RP, Enale H, Rajendran V, Mohan AMV (2020). Effect of positive electrode modification on the performance of zinc–bromine redox flow batteries. J. Energy Storage.

[124] Jeon JD, Yang HS, Shim J, Kim HS, Yang JH (2014). Dual function of quaternary ammonium in Zn/Br redox flow battery: capturing the bromine and lowering the charge transfer resistance. Electrochim. Acta.

[125] Wu MC, Zhao TS, Jiang HR, Zeng YK, Ren YX (2017). High-performance zinc bromine flow battery via improved design of electrolyte and electrode. J. Power Sources.

[126] Wu MC, Zhao TS, Zhang RH, Wei L, Jiang HR (2018). Carbonized tubular polypyrrole with a high activity for the Br_2_/B^−^ redox reaction in zinc–bromine flow batteries. Electrochim. Acta.

[127] Vogel I, Möbius A (1991). On some problems of the zinc–bromine system as an electric energy storage system of higher efficiency—I. Kinetics of the bromine electrode. Electrochim. Acta.

[128] Wang CH, Li XF, Xi XL, Xu PC, Lai QZ (2016). Relationship between activity and structure of carbon materials for Br_2_/Br^−^ in zinc bromine flow batteries. RSC Adv..

[129] Wu MC, Zhao TS, Wei L, Jiang HR, Zhang RH (2018). Improved electrolyte for zinc–bromine flow batteries. J. Power Sources.

[130] Hua L, Lu W, Li T, Xu P, Zhang H (2021). A highly selective porous composite membrane with bromine capturing ability for a bromine-based flow battery. Mater. Today Energy.

[131] Xu Q, Zhao TS (2015). Fundamental models for flow batteries. Prog. Energy Combust. Sci..

[132] Li MQ, Su H, Qiu QG, Zhao G, Sun Y (2014). A quaternized polysulfone membrane for zinc–bromine redox flow battery. J. Chem..

[133] Chakkaravarthy C, Waheed AKA, Udupa HVK (1981). Zinc–air alkaline batteries—a review. J. Power Sources.

[134] Tsehaye MT, Alloin F, Iojoiu C, Tufa RA, Aili D (2020). Membranes for zinc–air batteries: recent progress, challenges and perspectives. J. Power Sources.

[135] Kim R, Kim HG, Doo G, Choi C, Kim S (2017). Ultrathin nafion-filled porous membrane for zinc/bromine redox flow batteries. Sci. Rep..

[136] Grey CP, Tarascon JM (2017). Sustainability and in situ monitoring in battery development. Nat. Mater..

[137] Li M, Ran L, Knibbe R (2021). Zn electrodeposition by an in situ electrochemical liquid phase transmission electron microscope. J. Phys. Chem. Lett..

[138] Park JH, Steingart D, Koel B (2021). Visualizing zinc dendrites in minimal architecture zinc bromine batteries via in-house transmission X-ray microscopy. Microsc. Microanal..

[139] Kautek W, Conradi A, Fabjan C, Bauer G (2001). In situ ftir spectroscopy of the Zn–Br battery bromine storage complex at glassy carbon electrodes. Electrochim. Acta.

[140] Nolte O, Volodin IA, Stolze C, Hager MD, Schubert US (2021). Trust is good, control is better: a review on monitoring and characterization techniques for flow battery electrolytes. Mater. Horiz..

[141] Geiser J, Natter H, Hempelmann R, Morgenstern B, Hegetschweiler K (2019). Photometrical determination of the state-of-charge in vanadium redox flow batteries part I: in combination with potentiometric titration. Z. Phys. Chem. Int. J. Res. Phys. Chem. Chem. Phys..

[142] Kwabi DG, Wong AA, Aziz MJ (2018). Rational evaluation and cycle life improvement of quinone-based aqueous flow batteries guided by in-line optical spectrophotometry. J. Electrochem. Soc..

[143] Park S, Kim H, Chae J, Chang J (2016). Electrochemical generation of single emulsion droplets and in situ observation of collisions on an ultramicroelectrode. J. Phys. Chem. C.

[144] Xiao J, Li QY, Bi YJ, Cai M, Dunn B (2020). Understanding and applying coulombic efficiency in lithium metal batteries. Nat. Energy.

[145] Darling R, Gallagher K, Xie W, Su L, Brushett F (2015). Transport property requirements for flow battery separators. J. Electrochem. Soc..

[146] Aurbach D, Markovsky B, Weissman I, Levi E, Ein-Eli Y (1999). On the correlation between surface chemistry and performance of graphite negative electrodes for Li ion batteries. Electrochim. Acta.

[147] Xie CX, Li TY, Deng CZ, Song Y, Zhang HM (2020). A highly reversible neutral zinc/manganese battery for stationary energy storage. Energy Environ. Sci..

[148] Xie CX, Liu Y, Lu WJ, Zhang HM, Li XF (2019). Highly stable zinc–iodine single flow batteries with super high energy density for stationary energy storage. Energy Environ. Sci..

[149] Evans TI, White RE (1987). A review of mathematical-modeling of the zinc bromine flow cell and battery. J. Electrochem. Soc..

[150] Holmes J, White RE, Grimes PG, Bellows RJ, White RE (1984). Electrochemical Cell Design.

[151] Koo B, Lee D, Yi J, Shin CB, Kim DJ (2019). Modeling the performance of a zinc/bromine flow battery. Energies.

[152] Shah AA, Al-Fetlawi H, Walsh FC (2010). Dynamic modelling of hydrogen evolution effects in the all-vanadium redox flow battery. Electrochim. Acta.

[153] Schneider M, Rajarathnam GP, Easton ME, Masters AF, Maschmeyer T (2016). The influence of novel bromine sequestration agents on zinc/bromine flow battery performance. RSC Adv..

[154] Jin SJ, Jing Y, Kwabi DG, Ji YL, Tong LC (2019). A water-miscible quinone flow battery with high volumetric capacity and energy density. ACS Energy Lett..

[155] Sundén B (2019). Hydrogen, Batteries and Fuel Cells.

